# Autistic Traits and Camouflaging: A Meta-Analysis

**DOI:** 10.1177/13623613261437500

**Published:** 2026-04-24

**Authors:** Lachlan Greig, Sarah P Coundouris, Julie D Henry

**Affiliations:** 1 School of Psychology, The University of Queensland, Australia

**Keywords:** autism, camouflaging, masking, mental health

## Abstract

Autistic people sometimes camouflage their behaviour to appear non-autistic. This meta-analysis rigorously tests the relationship between autistic traits and camouflaging, examining contributing person- and study-level variables. Following Preferred Reporting Items for Systematic Reviews and Meta-Analyses (PRISMA) guidelines, we searched PubMED, PsycINFO, Web of Science, and ProQuest Dissertations in April 2025. All quantitative designs examining autistic traits and camouflaging on a continuum were included. Fifty papers (*N* = 16,895; ages 10–90) contributed to a three-level meta-analysis (accounting for dependent effect sizes). Study quality was medium to high with no evidence of publication bias. Results revealed a moderate association between autistic traits and camouflaging (*r* = 0.34, 95% CI: 0.30–0.39), comparable across sexes. Depression, but not anxiety or social anxiety, moderated the relationship. Age was not a moderator, but the association was stronger in general population samples (vs diagnosed), with self-reported autism measures (vs observational), and when using the discrepancy method for camouflaging (vs self-report). Among camouflaging subdomains, assimilation showed the strongest effect, followed by compensation and masking. Limited sample diversity constrains inferences across the full spectrum. This meta-analysis provides a clearer understanding of when, why, and how autistic traits are related to camouflaging, with important research and clinical implications. No funding was obtained for this study. Registration: https://osf.io/uswtr/?view_only=277aec07cdfc402dae75f4900f291253

**Lay Abstract:**

Understanding the autistic trait and camouflaging relationship is critical to identify who is most vulnerable to camouflaging and the way in which autism and camouflaging measurement may influence our understanding of this phenomenon. This directly impacts clinical diagnosis and support, as camouflaging contributes to diagnostic delay and poorer mental health outcomes, creating a cycle of continued camouflaging. Our findings may help to establish the foundation needed to develop targeted interventions.

We completed a systematic search to identify all studies that assessed the relationship between autistic traits and camouflaging. In total, 50 studies met all inclusion criteria. The first author extracted data related to participant characteristics (age, gender, diagnostic status, mental health), autistic trait characteristics, and the camouflaging measurement characteristics.

The 50 contributing studies included a total of 16,895 participants (61% female). These data show that the more autistic traits a person has, the more camouflaging they engage in; this relationship is evident for both males and females, and the strength of this relationship does not vary across the adult lifespan. People from the general population show an increase in the strength of this relationship, compared to those diagnosed, and the relationship changes based on how autistic traits and camouflaging are measured and conceptualised. Mental health did not have a clear impact on the overall relationship.

There is a nuanced relationship between autistic traits and camouflaging, the strength of which is dependent on specific person-related (diagnostic status and depression) and study-related factors (autistic trait measurement type, camouflaging measurement type, and camouflaging subdomain). Autistic traits are most strongly linked to behaviours that help people to assimilate (try to fit in and appear ‘normal’), followed by strategies to compensate for social differences. The act of hiding autistic traits was the least related. Because the relationship between autistic traits and camouflaging was weaker for diagnosed autistic people, further work is needed to test why this occurs. In addition, clinicians must be aware of the potential for camouflaging to disrupt the diagnostic process, and campaigns that aim to reduce stereotypes of autism and promote acceptance of neurodiversity may help to reduce the stigma that drives camouflaging.

## Introduction

Autism describes a diverse group of people who experience social communication difficulties and differences, repetitive behaviours, restricted interests, and/or sensory-related distress, which occur on a spectrum of impact and with commonly co-occurring conditions (American Psychiatric Association [APA], 2013; Lord et al., 2020). In recent years, research has grown among those who camouflage their autistic features and attempt to pass as non-autistic (Alaghband-rad et al., 2023; Allely, 2018). Camouflaging may occur consciously or subconsciously, and behaviours include imitation, forcing eye contact, excessive self-monitoring, researching and pre-preparing a social script, pretending to share others’ interests, developing different personas, hiding special interests, and repressing sensory coping strategies (Hull et al., 2020, 2021; Lei et al., 2023; Radulski, 2022; Tubío-Fungueiriño et al., 2021). While camouflaging is similar to impression management – the regulation of perceivable information to shape others’ view of the self (Ai et al., 2022; Miller et al., 2021) – the underlying motivation driving these behaviours is thought to differ: facilitating success for the general population, but social survival for autistic people (L. Bradley et al., 2021; Hull et al., 2017). Autistic people largely report that camouflaging occurs as a response to external pressure to fit in and to avoid discrimination and bullying. Some also suggest that they are motivated to achieve goals, and that camouflaging, and motivations for camouflaging, may change across contexts (Bernardin et al., 2021; Cage & Troxell-Whitman, 2019; Hull et al., 2017). These external pressures likely exist due to the stigmatisation, dehumanisation, and marginalisation of behavioural traits that have historically been pathologised or perceived as indicative of the underlying pathology (Pearson & Rose, 2021).

However, while qualitative reviews have reliably identified an association between autistic traits and increased camouflaging (Alaghband-rad et al., 2023; J. Cook et al., 2021), no quantitative integration has been conducted, meaning that the magnitude, robustness, and potential sources of heterogeneity in effects remain unclear. Consequently, the present article reports the first meta-analytic investigation into autistic traits and camouflaging to quantify the overall strength of this relationship and to test the potential moderating roles of person and study-related variables. In line with prior literature, our first hypothesis was that greater autistic traits would be associated with increased camouflaging.

### A Focus on Autistic Traits and Camouflaging

A trait-based approach to camouflaging is fundamental because it anchors the phenomenon empirically to the core characteristics of autism. Without establishing this foundational relationship, research on camouflaging risks operating on a premise that is assumed rather than rigorously tested. Therefore, by starting with autistic traits, the present study aimed to proactively build a model of the emergence of camouflaging, based on a key and measurable dimension of neurodiversity. Furthermore, given that camouflaging involves hiding autistic traits (Hull et al., 2017) which may disrupt diagnostic accuracy, and because broader systemic barriers can also impede access to a diagnosis (Malik-Soni et al., 2021; Moseley et al., 2018), we chose to focus on autistic traits rather than restrict our sample to formally diagnosed individuals. This trait-based approach also offers a more accurate and inclusive neurodiversity-informed understanding of camouflaging and how individuals adapt to social environments.

It is important to recognise, however, that transdiagnostic approaches emphasise the multi-dimensional nature of psychopathology and highlight limitations of diagnostic labels, such as arbitrary cutoffs, heterogeneity within diagnostic groups, and symptom overlap between diagnostic groups (Astle et al., 2021; Chetcuti et al., 2025). Indeed, a comparison of autistic and non-autistic groups reveals that autism has shared underlying features with other psychopathologies (Rodriguez-Seijas et al., 2019), raising the possibility that measures of autistic traits capture features across multiple conditions (Nenadić et al., 2021; Retzler & Retzler, 2023). However, even if such measures are not uniquely specific to autism, they remain valuable tools for capturing dimensions of neurodiversity relevant to camouflaging. Their utility may lie less in diagnostic delineation, but more so in capturing broader socio-cognitive patterns that help explain when, why, and for whom camouflaging emerges.

Finally, while much of the broader literature understandably focuses on potential mental health consequences of camouflaging, it is possible that this approach is limited by its reactive nature. A reactive approach considers camouflaging by examining its negative consequences. This is vital work, but it often means the phenomenon is only identified after distress has occurred, in part because camouflaging can occur subconsciously (Chapman et al., 2022) and can go undetected until poor mental health emerges (Pearson & Rose, 2021). By establishing correlates of camouflaging at the trait-level (including person and study-related moderators) it may be possible, with longitudinal designs, to develop models for identifying vulnerability before the cycle of camouflaging and its negative mental health outcomes become entrenched.

### Person-Related Factors

Females are widely thought to camouflage more than males (Allely, 2018; Tubío-Fungueiriño et al., 2021), and in the only study to date that directly compared the magnitude of the association between camouflaging and autistic traits across genders, the effect size increased from medium to large for females compared to males, respectively (Cardon et al., 2023). This finding lends support to the theory of a Female Autism Phenotype (Hull et al., 2020), which posits that, among potentially more diverse presentations of autistic traits, camouflaging may form a part of a unique female autistic experience. In line with this, our second hypothesis was therefore that the association between autistic traits and camouflaging would be stronger for females than for males.

Not only do autistic traits influence development throughout the lifespan (Wozniak et al., 2016), but the presence and severity of these traits fluctuate over time (Kroncke et al., 2016). Consequently, chronological age may also be important to understanding how autistic traits and camouflaging are related, and in the present review, no restrictions were placed on the age of included samples. Consistent with this idea, broader literature shows that the desire to fit in is not invariant across the lifespan (Nikitin & Freund, 2018) and that internalisation of stigma is also likely to change developmentally. However, to date, few studies have directly assessed the role of age in the relationship between autistic traits and camouflaging. In one notable exception, a study that included participants aged between 10 and 87 years identified a large, positive association between autistic traits and camouflaging for those over 15 years, but only a moderate, positive association for those younger than 15 years (Lundin Remnélius & Bölte, 2023). However, another age heterogeneous study of participants aged between 30 and 84 years identified a greater effect size for younger participants (van der Putten et al., 2023). Given this lack of clarity, the analysis was pre-registered with no *a priori* predictions made in respect to the nature of this effect.

As mentioned previously, broadening the scope to the general population may also capture those who camouflage but remain undiagnosed (Cook et al., 2021; McQuaid et al., 2022), thereby offering a more comprehensive understanding of camouflaging across the autistic trait continuum. This is particularly important given evidence that autistic traits are continuously distributed in the population and that subclinical traits can have meaningful psychosocial impacts (Chetcuti et al., 2025).

At present, it remains unclear whether receiving a diagnosis influences the process of linking autistic traits to camouflaging, and if so, in which direction. On the one hand, stigma associated with autism may encourage diagnosed individuals to camouflage; on the other, diagnosis may foster greater self-understanding and acceptance, potentially reducing the perceived need to camouflage (Wilson et al., 2023). Of the five studies that have tested whether the relationship between autistic traits and camouflaging differs as a function of diagnostic status, mixed results have been found. While most have identified a significant correlation across both cohorts (Bemmouna et al., 2023; Cassidy et al., 2021; Milner et al., 2023; Robinson et al., 2020; although see Belcher, 2020), one study identified a stronger correlation for autistic participants (Cassidy et al., 2021), but for three of these studies, this effect was stronger for the general population (Bemmouna et al., 2023; Milner et al., 2023; Robinson et al., 2020). Due to these mixed findings, as well as the lack of theoretical clarity on the mechanisms underpinning these effects, no hypothesis was made regarding the pre-registered sub-analysis of diagnostic status.

Finally, it is widely accepted that camouflaging is associated with poorer mental health (Evans et al., 2024; Hull et al., 2021; Leaf et al., 2023). While the links between mental health and camouflaging have been extensively studied, no study to date has conducted a specific test of the potential moderating role of mental health in the relationship between autistic traits and camouflaging. Theoretically, there are strong grounds for anticipating that depression, anxiety, and/or social anxiety may function as a moderator. As noted, autistic traits are stigmatised and frequently socially rejected (Pearson & Rose, 2021; Sasson et al., 2017), and those who are most sensitive to this social rejection, more acutely aware of social differences, or most self-critical may not only present with poorer mental health but also feel a stronger need to camouflage their autistic traits to prevent future social rejection. Indeed, depression, anxiety, and social anxiety have all previously been shown to be associated with camouflaging (Hull et al., 2021), but in this instance, we tested a model of interaction. Here, we therefore examined whether mental health changes the relationship between autistic traits and camouflaging, with our third hypothesis being that depression, anxiety, and/or social anxiety would moderate the association between autistic traits and camouflaging.

### Study-Related Factors

Autistic traits may be measured via self-report or observation. The most common self-report autistic trait measure – the Autism Quotient (AQ; Baron-Cohen et al., 2001) – assesses the presence of traits associated with the autism spectrum, while the most widely used observational measure – the Autism Diagnostic Observation Schedule (ADOS; Lord et al., 1989) – involves rating observable behaviour. Only one study to date has directly compared these two methods of assessment (Hannon et al., 2023), and this revealed that camouflaging was only related to autistic traits as indexed via self-report, and not via observational ratings. Given the limited data on this topic, this analysis was pre-registered without *a priori* predictions but simply sought to clarify whether the relationship between autistic traits and camouflaging differs as a function of how autistic traits are operationalised.

Different methods are also used to assess camouflaging behaviour, with the most frequently used approach being self-report, such as via The Camouflaging Autistic Traits Questionnaire (CATQ; Hull et al., 2019). This approach differs substantively from the discrepancy method (Lai et al., 2017), which involves standardising the scores on an observation and self-report autistic trait measure and calculating their discrepancy. The logic of this approach is that the greater the discrepancy between how autistic one appears behaviourally and the features of autism one reports, the greater the engagement in camouflaging. The only prior study to directly test whether the operationalisation of camouflaging influences its relationship to autistic traits found evidence consistent with this possibility (Hannon et al., 2023). However, given the limited prior empirical research testing this question, again, our pre-registered analyses here were exploratory.

Finally, it remains to be established whether specific aspects of camouflaging contribute differentially to this relationship. The CATQ distinguishes between three camouflaging subdomains: masking (hiding autistic traits), compensation (strategies used to overcome difficulties), and assimilation (attempting to fit in; Hull et al., 2019). Only three studies to date have considered the relationship between autistic traits and camouflaging at this sublevel, with two revealing positive correlations with compensation and assimilation, but not masking (Moore et al., 2023; Nieradka & Kossewska, 2023), and the third finding positive associations for all three, but of a lesser magnitude for masking (O’Loghlen & Lang, 2024). These findings therefore provide preliminary support for the idea that the relationship with autistic traits differs across different types of camouflaging behaviour, and again this analysis was pre-registered without a specific hypothesis being made.

### The Present Study

This study represents the first meta-analysis to quantify the relationship between autistic traits and camouflaging and the potential roles of person and study-related variables in understanding this relationship. This review therefore aims to systematically identify factors that are strongly linked to camouflaging, establishing a foundational evidence base for understanding who is most likely to engage in it, and under what methodological conditions this relationship is most clearly observed. The central pre-registered hypotheses were:

1. Greater autistic traits would be related with greater camouflaging.
2. Gender would moderate the relationship between autistic traits and camouflaging, such that the effect would be stronger for females relative to males.
3. Mental health would moderate this relationship, such that the effect size would be stronger for those with increased symptoms of depression, anxiety, and/or social anxiety.

## Method

The aims, hypotheses, and method of this meta-analytic review were pre-registered on the Open Science Framework (https://osf.io/uswtr/?view_only=277aec07cdfc402dae75f4900f291253) and adhered to PRISMA guidelines (Moher et al., 2009). The PRISMA checklist is reported as Supplementary 1.

### Search Strategy

A systematic literature search of titles and abstracts within PubMED, PsycINFO, Web of Science, and ProQuest Dissertations was conducted in April 2025. The search terms included clusters representing autism/autistic traits (autis* OR asd OR asperger*) and camouflaging (mask* OR compensat* OR camouflag*). The specific search strategy employed was (autis* OR asd OR asperger*) AND (mask* OR compensat* OR camouflag*). This was supplemented by a backward citation search (Alaghband-rad et al., 2023; Allely, 2018; J. Cook et al., 2021; Libsack et al., 2021; Tubío-Fungueiriño et al., 2021); however, no additional novel studies were identified. During the search, papers published in a language other than English were translated by Google Translate. In instances where the necessary data for analyses were not reported in the publication, we contacted the corresponding author via email. We sent one follow-up email to all authors who did not respond to the first contact. When the publication was more than 10 years old, we did not contact the author. There were no restrictions placed on the age of included samples.

### Search Eligibility Criteria

**Figure 1.**
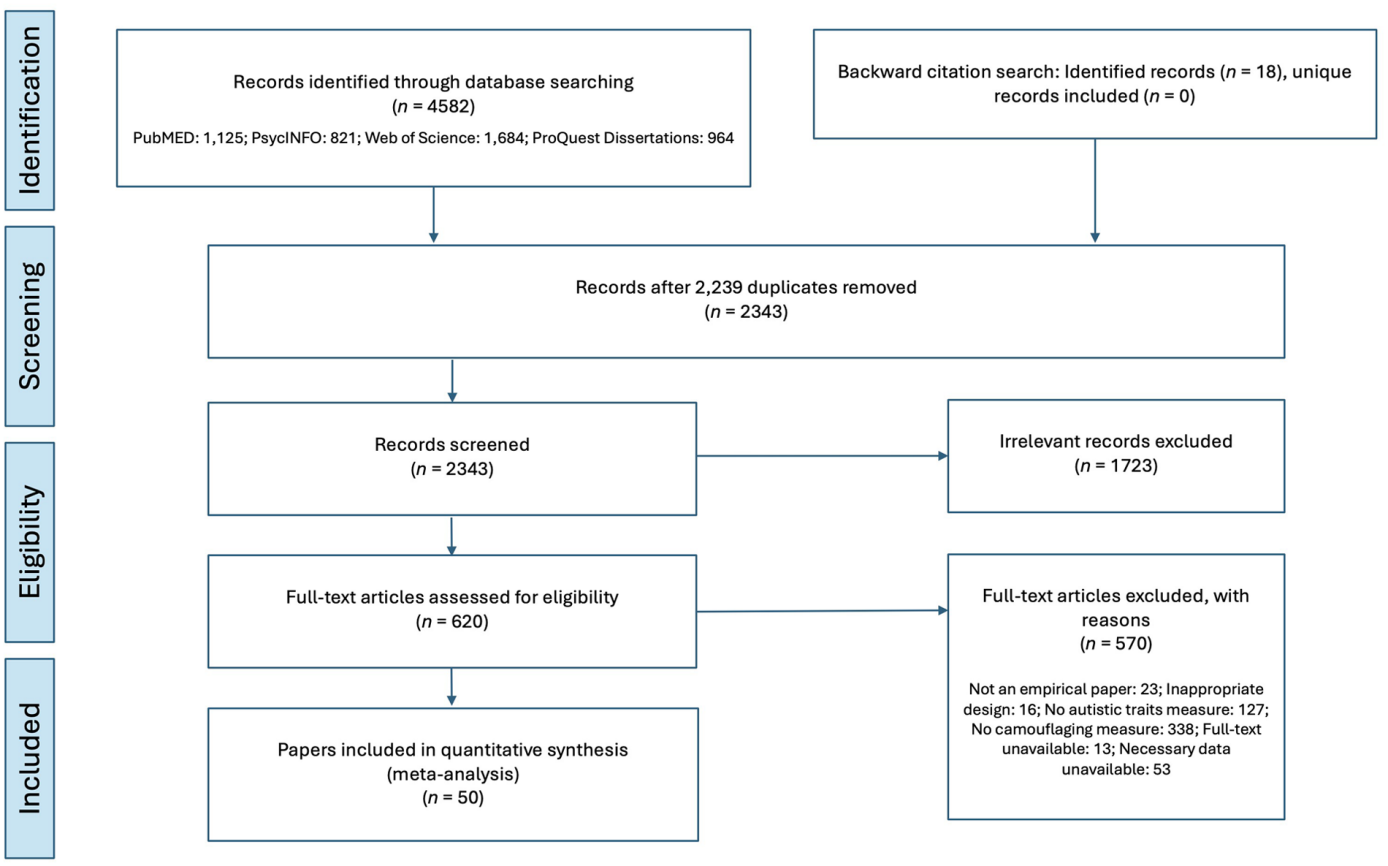
PRISMA Flowchart of Study Screening and Selection Process.

All titles and abstracts were initially screened for ineligibility by the first author, and a second rater checked agreement on 20% of studies to ensure suitable inter-rater reliability (pre-registered as Cohen’s *K* = 0.85; inter-rater reliability achieved = 0.86). In instances of uncertainty, the two raters resolved the discrepancy together. Full text screening was completed by the first author, and a second rater checked 10% of studies (interrater reliability *K* = 1.00). The PRISMA study screening and selection process is shown in Figure 1. Studies were considered eligible if they:

1. Were quantitative studies that report original data;
2. Used at least one continuous measure of autistic traits;
3. Used at least one continuous measure of camouflaging; and
4. The statistics were published or provided by the author upon request and could be used to calculate a precise relationship effect size.

### Definitions of Variables

#### Autistic Traits

Any scale solely measuring autistic traits on a continuum, in formally diagnosed autistic people and/or general population, were permitted to contribute. Further coding was used to specify whether autistic traits were measured through self-report or observation. *Self-report* asks participants to rate their own agreement, with the most common self-report scale being the AQ (Baron-Cohen et al., 2001). Alternatively, *observation* involves a semi-structured interview and/or monitoring of behaviour in naturalistic settings, by a trained health professional or researcher (e.g. ADOS; Lord et al., 1989), or a parent-report measure of autistic traits whereby parents reflect on their child’s autistic traits (e.g. AQ-10-A; Allison et al., 2012).

#### Autistic Camouflaging

Autistic camouflaging broadly refers to hiding autistic traits to avoid stigma, judgement, or to attempt to fit in (Hull et al., 2020). In the literature, this term has been used interchangeably with masking, compensation, or passing as non-autistic. Here, camouflaging was considered as the overarching construct, with the three camouflaging subtypes – *masking* (e.g. I monitor my body language or facial expressions so that I appear relaxed), *compensation* (e.g. When I am interacting with someone, I deliberately copy their body language or facial expressions), and *assimilation* (e.g. In social situations, I feel like I’m ‘performing’ rather than being myself) – also coded where possible (Hull et al., 2019). Two broad types of methodological approach were used to operationalise camouflaging: *self-report* that measures the extent to which a person reports the use of camouflaging, and *discrepancy* that reflects the discrepancy between a person’s internal autistic state and their observable autistic traits (e.g. the discrepancy between the AQ and the ADOS).

#### Mental Health

Mental health was operationalised as depression, anxiety, and/or social anxiety. Any continuous scale that measures depression, anxiety, and/or social anxiety symptoms and provides an individual score for depression, anxiety, and/or social anxiety was included. All measures included were multi-item self-report scales. There were no instances in which multiple symptom groups were collated under an overall mental health label, and thus, in each case, we were able to assess depression, anxiety, and social anxiety separately. Depression is clinically defined by a depressed mood and may include a negative affect, decreased energy, weight loss, sleep disturbance, feelings of worthlessness, and sometimes suicidal ideation (APA, 2013). Anxiety is clinically defined by excessive worry, and symptoms include fatigue, difficulty concentrating, irritability, and sleep disturbance (APA, 2013). Anxiety measures encompassed generalised anxiety disorder, broader anxiety, and autism-specific anxiety. Social anxiety is clinically defined by intense fear of social scrutiny (APA, 2013).

### Quality Assessment

In line with PRISMA recommendations, to assess the quality of each individual study, the appraisal tool for cross-sectional studies was used (AXIS; Downes et al., 2016) and is reported in the Quality Assessment section. This tool typically involves 20 criteria; however, in the current review, three questions were removed *a priori* due to difficulty in applying these criteria to samples generally recruited online, as was the case for some studies contributing to this meta-analysis. While the AXIS does involve subjective judgement, this has been identified as a strength because it allows for greater flexibility (Downes et al., 2016). Decisions are made dichotomously, where yes is classified as one, and no/unclear is classified as zero. Here, studies were assessed within the subcategories reporting, design, and potential biases, and studies were classified low, medium, or high quality (low: 0–3 for reporting and design, 0–1 for biases; medium: 4–5 for reporting and design, 2 for biases; high: 6–7 for reporting and design, 3 for biases; question 19 reverse scored). When a paper reported multiple studies (Dodd, 2022), each relevant study was assessed separately.

### Data Extraction

Data extraction and coding was conducted by the first author. A second rater checked agreement on 25% of included studies. The following data were extracted:

1. Participant characteristics: age, gender, diagnostic status (autistic or general population).
2. Autistic trait characteristics: the method of measuring autistic traits (self-report or observation) and measure(s) used (e.g. AQ, ADOS).
3. Autistic camouflaging characteristics: the method(s) of measuring camouflaging (self-report or discrepancy), measure used (e.g. CATQ), and in the case of the CATQ, the subdomain (e.g. masking, compensation, or assimilation).
4. Depression, anxiety, and social anxiety: measure(s) used and mean scores. All studies included measured or scored these constructs independently, and therefore, we were able to keep each separate, rather than aggregating into an overall construct representing mental health.
5. Relevant statistics to calculate precise effect sizes.

### Statistical Analyses

To analyse data, the *metafor* (Viechtbauer, 2010), *plyr* (Wickham, 2011), *Hmisc* (Harrell, 2024), *readxl* (Wickham et al., 2023), *dplyr* (Wickham & Bryan, 2023), *dmetar* (Harrer et al., 2019), and *QuantPsyc* (Fletcher, 2022) packages were used in RStudio (version 2024.04.2; RStudio Team, 2020). Analyses were conducted using Fisher’s *Z* and then transformed to *r* values for interpretability. Following Cohen’s (1977) interpretation of *r*, effect sizes of 0.10 were considered small, 0.30 moderate, and 0.50, or greater, as large. A positive relationship suggests that higher autistic traits relate to increased camouflaging. All effect sizes were derived only from correlations. Conversion of effect sizes from regressions was deemed acceptable, but no study with missing correlational data provided data in this format.

As most studies reported multiple outcomes, a three-level random-effects meta-analytic model was used to account for the dependency between effect sizes (Cohen, 1977). Three-level models reduce the potential for Type 2 error by avoiding averaging effect sizes together and allow precise estimation of variance at each level (within-study and between-study; Assink & Wibbelink, 2016). The models were fitted using the Restricted Maximum Likelihood (REML) estimator. The proportion of variance (*I*^2^) at levels 2 and 3 and the 80% prediction interval for future studies were calculated. Separate moderator (and subgroup for categorical variables) analyses were conducted for person (gender, age, diagnostic status, mental health), and study-related variables (autistic trait measurement type and specific measure, camouflage measurement type, and camouflage subdomain). Mental health was considered separately for depression, anxiety, and social anxiety via multivariate moderator analyses to address measure variability (measure used and mean score). All research questions, both hypothesised and exploratory, were pre-registered on Open Science Framework (https://osf.io/uswtr/?view_only=277aec07cdfc402dae75f4900f291253).

Deviations from the original pre-registration plan are documented on the Open Science Framework and outlined below. The analysis of camouflaging measurement type (self-report and discrepancy) was updated on June 14, 2024, to consider the relationship with self-reported and observed autistic traits separately. This was due to the likelihood that the relationship would differ between types of autistic trait measurement (e.g. discrepancy measures of camouflage would likely interact with observed and self-reported autistic traits in different ways) and therefore potentially obscure the true strength and direction of the relationship.

We conducted a post hoc quadratic moderator analysis of age (26th March 2025) because the two existing studies focusing on age, when considered together, suggested a non-linear pattern, with camouflaging potentially being more prominent in young adulthood than in both childhood and older adulthood (Lundin Remnélius & Bölte, 2023; van der Putten et al., 2023). We conducted two further post hoc age analyses (18th November 2025) because the age range in our meta-analysis was predominantly representative of adulthood. For this reason, we ran the analyses omitting all child samples. In addition, we conducted a categorical moderator analysis comparing adult-only samples and child-only samples.

Social anxiety was not pre-registered as a potential moderator variable in Hypothesis 3 but was later included (14th April 2025) based on peer-review feedback. We initially chose to focus on generalised anxiety due to the greater amount of data available compared to social anxiety, but given that our mental health hypothesis was centred around social rejection and social awareness, and we did have enough data to analyse social anxiety too, it made the most sense to include both.

To assess the impact of publication bias (Nair, 2019), Orwin’s Failsafe *N* (Orwin, 1983), Egger’s regression test (Egger et al., 1997), and trim-and-fill analyses were used. Two sensitivity techniques, Cook’s distances (R. D. Cook & Weisberg, 1982) and standardised residuals (scores with ±2.24 standard deviations are considered outliers; Aguinis et al., 2013), were used to assess if any effect sizes were exerting disproportionate influence. A new summary effect was calculated excluding each individual case to assess any potential significant change (Viechtbauer & Cheung, 2010), as well as a new summary effect while excluding all outliers, and a new summary effect while excluding all related outliers (e.g. discrepancy method outliers).

## Results

### Quality Assessment

**Figure 2.**
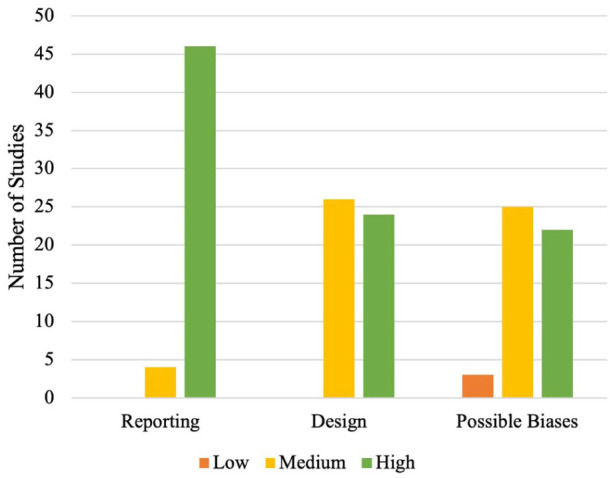
AXIS Subcategory Quality Classifications.

Most studies were classified as medium or high quality, across the three sub-categories, as shown in Figure 2. Importantly, no study was classified as poor quality across multiple sub-categories, with only three studies rated poor quality on one sub-category, possible biases.

**Figure 3.**
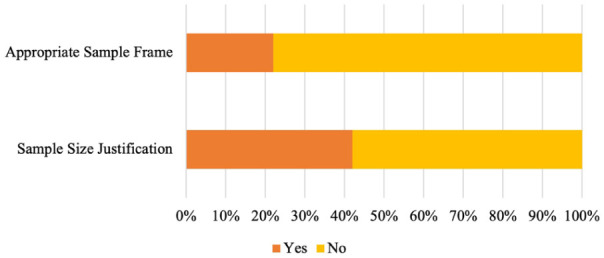
AXIS Criterions Met by Less Than Half of Included Studies.

An examination of each criterion revealed that only two of the criteria were met by less than 50% of the studies appraised (see Figure 3). First, only 22% of studies used an appropriate sample frame. That is, studies frequently reported an uneven gender split (most commonly a female majority), and/or the sample mostly comprised participants with limited diversity (age, race), a high IQ or education attainment, and/or a diagnosis in adulthood. In other cases, the basic data reported was insufficient to make an accurate assessment of the sample frame. Control groups or studies of autistic traits in the general population were frequently convenience samples of university students. Second, only 42% of studies justified the sample size used in analyses, and while most studies appeared to have sufficient sample sizes, without justification such as an *a priori* power analysis, studies could be at risk of being underpowered to detect certain key effects. Taken together, while most studies were medium or high quality, limitations of the sample frame should be considered when evaluating the literature.

### Summary Characteristics

In total, 50 papers contributed to the dataset, comprised of 51 studies, 112 unique samples, 298 individual effect sizes, and 16,895 participants (see Table 1 for a full breakdown). Most samples were from the UK (39%), followed by the USA (14%), ages ranged from 10 to 90 years (*M* = 31.42, pooled *SD* = 9.49), and 61% of the total sample were female. Forty percent of samples were formally diagnosed, 9% were a combination of formally and self-diagnosed, 40% were from the general population, and 11% did not differentiate participants by diagnostic status.

**Table 1.** Participant Summary Characteristics.

Characteristic	k	Characteristic	*n*
**Country**		**Sample size**	17,327
UK	44	**Gender**	
USA	16	Male	6,058
Poland	11	Female	10,627
Japan	10	Non-binary/other	642
Australia	6	**Age *M (SD)***	31.42 (9.49)
Canada	6	**Adult samples %, *M (SD)***	92%, 32.51 (8.91)
China	3	**Child samples %, *M (SD)***	6%, 14.67 (1.40)
France	2	**Mixed age samples %, *M (SD)***	2%, 31.45 (16.91)
Taiwan	2		
Netherlands	1		
Italy	1		
Mixed	10		
**Diagnostic status**			
Autistic (formally diagnosed samples)	45		
Autistic (formally and self-diagnosed samples)^a^	10		
General population	45		
Mixed^b^	12		

a Autistic (formally and self-diagnosed samples) did not contribute to the main diagnostic status moderation but are included in the autistic group in Supplementary 4. ^b^ Mixed samples did not contribute to the diagnostic status moderation analysis.

### Meta-Analytic Results

**Figure 4.**
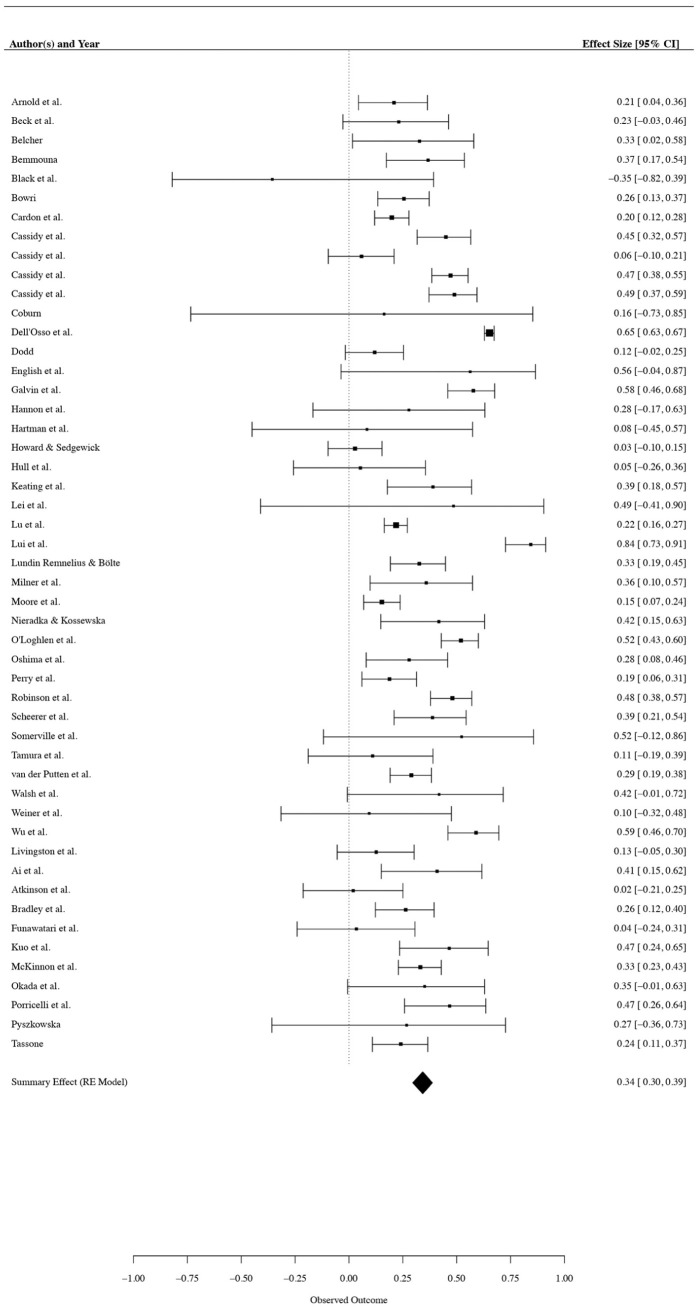
Forest Plot Displaying Effect Sizes, Confidence Intervals, and Weights for Studies Contributing to the Summary Effect Size.

Aggregated across 298 effect sizes, a moderate, positive association was found between autistic traits and camouflaging (*r* = 0.34, 95% CI: 0.30–0.39, *p* < 0.001), such that greater autistic traits were associated with increased camouflaging. The estimated variance components were *I*^2^_*level2*_ = 95.28 (within-study variance) and *I*^2^_*level3*_ = 0.23 (between-study variance), meaning that most variance was reported at the within-study level and not attributable to sampling error. In line with the significant heterogeneity identified, the prediction interval suggests with 80% likelihood that an effect size of a new study would span from a large positive effect to a small negative effect (*r* = −0.17 to 0.71). A forest plot displaying effect sizes, confidence intervals, and weights is shown in Figure 4, and a detailed summary of participant and study characteristics is reported as Supplementary 2.

There was no evidence of publication bias, Orwin’s Failsafe *N* = 1826, Egger’s Regression Test = −0.92, and Trim and Fill imputed *r* = 0.36. Sensitivity analyses identified nine outlier effect sizes, six of which used a discrepancy camouflaging measure. We ran the analyses while excluding each outlier individually and while excluding all outliers at once; however, as the summary effect was unchanged by exclusion, the original dataset was retained for further analyses.

#### Moderator Analyses

Table 2 reports the results of each categorical moderator analysis.

**Table 2.** Outcomes of Categorical Moderator Analyses.

		95% CIs						
Contrast	*r*	Lower	Upper	*PVAF*	*K*	*k*	*ES*	*N*
**Gender**								
Female	0.36***	0.29	0.43	12.96	28	33	109	3,839
Male	0.32***	0.20	0.46	10.24	27	33	97	2,788
**Age**								
Adult	0.34***	0.18	0.61	11.56	56	101	257	16,028
Child/Adolescent	0.34***	0.18	0.52	11.56	3	9	35	228
Diagnostic status**								
Autistic	0.26**	0.15	0.37	6.76	21	45	117	3,179
General population	0.42***	0.35	0.48	17.64	22	45	124	9,201
Autistic trait measure type**								
Self-report	0.36***	0.18	0.52	12.96	49	111	274	16,823
Observation	0.01	−0.18	0.20	0.01	6	10	24	336
**Autistic trait measure**								
AQ variants	0.32***	0.26	0.37	10.24	35	78	172	10,153
Other	0.35***	0.23	0.47	12.25	15	36	97	5,424
Camouflage measure type (association with self-reported autistic traits)***								
Self-report	0.33***	0.29	0.38	10.89	47	107	266	16,645
Discrepancy	0.92***	0.87	0.96	84.64	4	8	8	206
Camouflage measure type (association with observed autistic traits)*								
Self-report	−0.37	−0.73	0.15	13.69	4	6	16	136
Discrepancy	0.25*	−0.24	0.64	6.25	4	8	8	206
Camouflage subdomain***								
Masking	0.10**	0.03	0.18	1.00	26	68	79	8,369
Compensation	0.37***	0.30	0.43	13.69	26	68	79	8,369
Assimilation	0.50***	0.44	0.55	25.00	27	69	81	8,546

*PVAF* = percentage of variance accounted for. *K* = number of studies. *k* = number of independent samples. *N* = number of participants contributing to the effect. A positive effect size represents increased camouflaging.

* *p* < 0.05. **p < 0.01. ***p < 0.001.

##### Person-Related Factors

Gender did not emerge as a significant moderator, *F*(1,247) = 0.53, *p* = 0.466, with comparable effect sizes for males and females (see Table 2) and significant heterogeneity remaining, *Q* = 3004.67, *p* < 0.001. A moderation analysis, with gender non-conforming participants contributing, is reported in Supplementary 3.

Age, measured continuously, also did not moderate the relationship, *b* = −0.00, 95% CI [−0.01, 0.01], *k* = 89, *F*(1,231) = 0.03, *p* = 0.858, with significant heterogeneity remaining, *Q* = 3193.48, *p* < 0.001. A post hoc quadratic moderator analysis was also non-significant, *b* = −0.00, 95% CI [−0.00, 0.00], *k* = 89, (*F*(2,230) = 0.02, *p* = 0.981) suggesting that the relationship between autistic traits and camouflaging is robust across the lifespan. Due to the relatively small number of child/adolescent samples compared to adult samples, we re-ran the overall model only including adult-only samples; this did not impact the outcome of the main analysis (*r* *=* 0.34, 95% CI: 0.29–0.39, *p* < 0.001). We also conducted a categorical moderator analysis to compare adult-only and child/adolescent-only samples, but the moderation model was non-significant, *F*(1,290) = 0.04, *p* = 0.950, and again significant heterogeneity remained *Q* = 4429.35, *p* < 0.001.

Diagnostic status did emerge as a significant moderator, *F*(1,261) = 8.85, *p* < 0.001, with substantial remaining heterogeneity, *Q* = 4024.57, *p* < 0.001. While the relationship was significant for formally diagnosed autistic and those in the general population, the strength of this relationship increased from moderate to large for those in the general population (Table 2). A moderation analysis with self-diagnosed autistic participants included in the diagnosed group is reported in Supplementary 3.

While the multivariate depression model was significant, *b* = −0.04, 95% CI [−0.10, 0.02], *k* = 49, *F*(15,108) = 1.79, *p* = 0.045, the anxiety and social anxiety models were non-significant, *b* = −0.03, 95% CI [−0.10, 0.04], *k* = 44, *F*(11,99) = 1.03, *p* = 0.429, and *b* = −0.00, 95% CI [−0.01, 0.00], *k* = 26, *F*(4,71) = 0.742, *p* = 0.566, respectively. The models for depression, anxiety, and social anxiety had substantial remaining heterogeneity after accounting for each variable, *Q* = 1632.59, *p* < 0.001, *Q* = 1655.99, *p* < 0.001, and *Q* = 833.60, *p* < 0.001, respectively.

##### Study-Level Factors

Autistic trait measurement type was a significant moderator, *F*(1,296) = 13.55, *p* < 0.001, *Q* = 4426.61, *p* < 0.001, such that the relationship between autistic traits and camouflaging only held when autistic traits were measured via self-report (see Table 2). Significant heterogeneity was identified after accounting for autistic trait measurement, *Q* = 4426.61, *p* < 0.001. Furthermore, comparing the AQ plus variants to other self-reported measures was non-significant, *F*(1,296) = 2.73, *p* = 0.100, and again, significant heterogeneity remained, *Q* = 4479.75, *p* < 0.001.

Camouflaging measurement type also emerged as a significant moderator when analysed separately for self-reported and observed autistic traits, *F*(1,272) = 74.25, *p* < 0.001, and *F*(1,22) = 7.18, *p* = 0.014, respectively. Specifically, as reported in Table 2, for self-reported autistic traits, while both methods were related to greater camouflaging, the size of this effect was moderate for self-report but large for discrepancy. For observed autistic traits, self-reported camouflage was non-significant, while the discrepancy method had a moderate positive relationship. Significant heterogeneity remained after accounting for camouflaging measurement type for self-reported and observed autistic traits, *Q* = 3860.87, *p* < 0.001, and *Q* = 144.69, *p* < 0.001, respectively.

Finally, camouflaging subdomain had a significant moderating effect *F*(2,236) = 69.30, *p* < 0.001, but with substantial remaining heterogeneity, *Q* = 2215.95, *p* < 0.001. Although the direction of the relationship did not change, the association was small for masking, moderate for compensation, and large for assimilation (Table 2).

## Discussion

Interest in understanding camouflaging of autistic traits has grown exponentially in recent years, in part because camouflaging one’s authentic self to fit in with the societal norms has been hypothesised to negatively impact receiving an autism diagnosis (J. Cook et al., 2021; Pearson & Rose, 2021) and broader wellbeing (Evans et al., 2024; Hull et al., 2021; Leaf et al., 2023). Accordingly, this review aimed to provide a comprehensive integration of extant literature and clarify which person and study-related factors are relevant to understanding the association between autistic traits and camouflaging.

The first aim of this meta-analysis was to assess the extent to which camouflaging is empirically linked to the autistic phenotype it is theorised to suppress. In line with pre-registered Hypothesis 1, greater autistic traits were associated with increased camouflaging. This finding is important in showing not only that this effect exists, but that it is moderate in magnitude and thus consistent with broader literature that suggests autistic traits are a robust, but not exclusive, correlate of camouflaging behaviour. One of the reasons why camouflaging is hypothesised to emerge is because of the stigmatisation of autistic traits (Miller et al., 2021), with autistic traits overwhelmingly rated negatively by observers (Pearson & Rose, 2021; Sasson et al., 2017). Since awareness of self-relevant external stigmatisation may lead to self-directed stigma (Riebel et al., 2024), it follows that, to protect the self from stigma, greater autistic traits would be associated with increased camouflaging. Furthermore, given that autistic traits were not an exclusive correlate of camouflaging, such behaviours may emerge more broadly from atypical social behaviour linked to other marginalised traits, consistent with transdiagnostic theories. However, the primary contribution of this work lies in moving beyond simply documenting this association exists, establishing which variables moderate how strongly it presents (discussed next), thereby establishing a foundation for understanding when and for whom camouflaging might be more pronounced.

### Person-Related Factors

Contrary to pre-registered Hypothesis 2, the relationship between camouflaging and autistic traits was not moderated by gender, and indeed, the magnitude of each group’s aggregate effect size was comparable (although confidence intervals suggest increased variance among males compared to females). This finding of gender parity is inconsistent with the only empirical study to date to directly compare males and females (Cardon et al., 2023) and, in part, contradicts a leading hypothesis in the literature. The Female Autism Phenotype (Allely, 2018; Hull et al., 2020) posits a unique female autistic profile of which camouflaging is a feature. Our results suggest that, rather than reflecting a distinct female-specific mechanism, camouflaging operates as a general response to autistic traits that is evident across genders. This does not preclude the possibility that autistic females engage in camouflaging more frequently; indeed, evidence suggests they do (Hull et al., 2020). However, it indicates that whatever drives higher average levels in females does so without altering the fundamental link between autistic traits and camouflaging behaviour. The current findings therefore add nuance to a potential female autism phenotype by highlighting that overall levels may be driven by a variable not tested or identified here. Furthermore, it is important to acknowledge that measures may be biased, such that typical male autistic behaviours are more likely to be detected (Belcher et al., 2023; J. Cook et al., 2021). Future studies are therefore needed to better understand both how autistic trait presentations as well as camouflaging may differ as a function of gender.

Chronological age (linear and quadratic) also failed to moderate the association between autistic traits and camouflaging. As noted, age has rarely been considered in broader camouflaging literature, and these findings therefore provide the strongest evidence to date that the relationship between camouflaging and autistic traits is age invariant, and that negative evaluations of autistic traits may be encountered throughout the lifespan (Mitchell et al., 2021). We also conducted post hoc control analyses to address the potential impact of the smaller number of child and adolescent samples. First, re-running the overall model using only adult samples yielded an identical result to the original model including all ages. Second, a categorical moderator analysis directly comparing child/adolescent-only samples to adult-only samples was non-significant. Taken together, these analyses suggest that the relationship between autistic traits and camouflaging is stable across the adult lifespan and does not differ significantly between childhood/adolescence and adulthood. However, it is critical to note that only a small number of the contributing studies included young people. This limited representation highlights an important gap in the literature, and further research is needed to better understand the developmental trajectory of camouflaging in childhood and adolescence.

Diagnostic status emerged as a significant moderator, with a substantially stronger association between camouflaging and autistic traits identified in general population compared to diagnosed samples. While further research is needed to directly establish what is driving this effect, several considerations warrant particular attention. First, lesser variance in autistic trait levels in the autistic group compared to the general population may have obscured the true strength of the association, meaning the between-group difference may not be as substantial as it appears. Future research is now needed to rule out this possibility. On the other hand, it could be that autistic traits are a stronger correlate of camouflaging engagement for individuals in the general population than for those diagnosed as autistic. The weaker relationship within the diagnosed groups could suggest that the psychological mechanism linking autistic traits to camouflaging may be altered post-diagnosis. Although some might view this interpretation as counterintuitive, it aligns with qualitative work suggesting that diagnosis can foster self-understanding and reduce the need to camouflage (Wilson et al., 2023). Furthermore, autistic trait measures may capture overlapping features of other camouflageable psychopathology, consistent with transdiagnostic theories. It would then follow that camouflaging may reflect a broader strategy in response to perceived social norms, rather than being autism exclusive. Finally, camouflaging measures may capture impression-management behaviours when used in the general population (Ai et al., 2024), with future studies needed to investigate whether camouflaging measurement can distinguish distinct between groups motivations. While self-diagnosis of autism is often considered valid (Coburn, 2021), many studies either only included those who reported a formal diagnosis (e.g. Arnold et al., 2023; Belcher, 2020) or combined formally diagnosed and self-diagnosed participants (e.g. Atkinson et al., 2025; S. Bradley et al., 2024), and therefore, we could not compare the effect between those formally diagnosed and self-diagnosed.

In partial support for pre-registered Hypothesis 3, symptoms of depression, but not of anxiety or social anxiety, moderated the overall association. It was anticipated that stigmatisation of autistic traits could result in poorer mental health and thereby motivate camouflaging to avoid the resulting negative consequences. However, the analysis of depression, although significant, revealed substantial heterogeneity suggesting that depression influences the relationship between autistic traits and camouflaging in a more complex manner than originally theorised.

The failure to identify any moderating role of anxiety or social anxiety was unexpected given the strong association between autism and poor mental health (Lai, 2023), and future research is needed to better understand these null results. However, it may be that camouflaging is associated with little variance in anxiety because it is a no-win situation, such that people who do not camouflage are rejected, yet people who do camouflage suppress their authentic selves (Evans et al., 2024). Alternatively, if mental health and autistic trait measures assess overlapping features, the limited moderating effect of mental health on camouflaging may reflect this conceptual redundancy rather than a true lack of influence. Given all included studies were cross-sectional, bar one, longitudinal studies are a particularly important next step in this literature to establish precisely how mental health and camouflaging are related.

### Study-Related Factors

Sub-group analyses revealed that only autistic traits measured via self-report were significantly associated with camouflaging, and this was consistent across different self-report instruments (AQ, Broad Autism Phenotype Questionnaire [BAPQ], Ritvo Autism & Asperger Diagnostic Scale – 14 [RAADS-14], etc.). The lack of association between camouflaging and observable autistic traits could reflect successful camouflaging, while self-report methods could also capture an underlying experience beyond what is observable to others. In addition, standard diagnostic tools could be impacted by measurement bias, whereby certain characteristics or presentations may be less likely to be identified. Given that the observation method is the standard diagnostic approach for autism, these findings highlight the need to carefully consider camouflaging during the diagnostic process.

Also speaking to the importance of measurement type, camouflaging measure also emerged as a moderator when considered separately for self-reported and observed autistic traits. For self-reported autistic traits, while a significant effect was evident for both camouflaging measurement types, it was greater for the discrepancy method. For observed autistic traits, a positive association only emerged for the discrepancy method, with no association evident for self-report. It has previously been suggested that the discrepancy method captures not simply a person’s intent to engage in camouflaging but also the efficacy with which they do so (Hannon et al., 2023). Taken together, these findings therefore suggest that it might not be engagement in camouflaging *per se* that is most sensitive to autistic traits, but rather successful and/or subconscious engagement. However, it is important to note that correlations involving discrepancy-based camouflaging measures share methodological overlap with the autistic trait measure used to calculate the discrepancy. This is because the same instrument contributes to both sides of the correlation (e.g. the AQ appears in both the autistic trait score and the camouflaging score when correlating AQ with AQ-ADOS). This shared variance may partly account for the larger effect sizes observed for this method compared to self-reported camouflaging.

Finally, although each specific camouflaging subdomain was associated with autistic traits, assimilation had the strongest effect, followed by compensation, and masking. For people with elevated autistic traits, their efforts are most strongly linked to behaviours that help them to try to fit in and appear ‘normal’, followed by strategies to compensate for social differences. The act of hiding autistic traits was the least related. Indeed, certain autistic traits, like sensory stimming, appear to serve important self-regulatory functions (Radulski, 2022), and so may be less frequently suppressed when doing so would increase distress. This preference for assimilation, may therefore reflect a desire to maintain beneficial coping mechanisms while still navigating social norms. This suggests some camouflaging behaviours may stem from more general socio-cognitive or emotional challenges, not exclusively from autistic traits, reflecting a broader, transdiagnostic adaptation to perceived social threats or pressures to conform.

### Limitations and Future Directions

As with any meta-analysis, confidence in the conclusions is dependent on the contributing data. The AXIS quality assessment identified that studies included in this review frequently reported samples with a female majority, limited diversity of age and ethnicity, high educational attainment, and/or a diagnosis in adulthood which therefore limits the generalisability of these findings to the entire autistic spectrum. By showing these biases exist across much of this literature, this meta-analysis will hopefully provide an impetus for future studies to better capture the full heterogeneity of those who camouflage, by recruiting more diverse samples. This must also include diverse and non-binary gender identities as literature suggests increased gender diversity among autistic people compared to the general population (Glackin et al., 2024).

It is also important to acknowledge that, despite the fact the overall relationship did not differ significantly when compared between AQ and non-AQ measures of autistic traits, the AQ is subject to various criticisms in this literature. Namely, this measure may be biased against females (Belcher et al., 2023) and only measures the presence, but not the severity of autistic traits (Baron-Cohen et al., 2001). Consequently, it remains possible that, after accounting for these limitations, this relationship could change. In addition, an important next step in this literature is to establish whether specific autistic traits or behaviours are more likely to be subject to camouflaging than others.

In addition to the limitations of the extant literature, several limitations of the present review and directions for future research should be noted. First, we combined studies using different methodologies (e.g. self-reported and observed autistic traits, self-reported camouflaging, and discrepancy camouflaging), and this likely added some noise to the data. Second, as a synthesis of cross-sectional data, our review can speak to association and moderation, but not to causality, mediation, or other psychological mechanisms (e.g. internalised stigma, social motivation). Third, despite the significant moderators identified, substantial heterogeneity remained unaccounted for. A multiple-moderation model including all significant variables was not possible to analyse because missing data for any included variable results in the exclusion of that effect size and thus yields a non-representative outcome. Therefore, it is not clear if moderator variables taken together may account for substantial heterogeneity. It is also possible that this heterogeneity reflects the multiple methods included in the review and/or the influence of additional person-level or study-level variables not captured in our analysis. Future research should continue to explore potentially relevant variables, such as self-compassion or internalised stigma (Ai et al., 2024; Galvin et al., 2024), to deliver a more complete model of the circumstances under which autistic traits and camouflaging are most strongly linked. Together, these points need to be considered when interpreting our findings and should be used to inform future research, moving towards a more complete model of the circumstances under which autistic traits and camouflaging are most strongly linked, as well as the possibility that camouflaging represents a transdiagnostic phenomenon extending beyond autism.

### Practical and Clinical Implications

The findings of this meta-analysis have important implications for autistic individuals, those with elevated autistic traits, and clinicians. First, the robust association between autistic traits and camouflaging suggests that individuals with higher autistic traits, regardless of age or gender, may benefit from increased awareness of camouflaging. Second, clinicians need to be cognisant of the potential for camouflaging to influence the diagnostic process given the lack of relationship with observable autistic traits identified in these data. To better understand the lived experience of clients, clinicians are encouraged to incorporate validated self-report measures of both autistic traits and camouflaging alongside traditional observational assessments. Third, the strong association between autistic traits and camouflaging in general population samples suggests that camouflaging is not unique to diagnosed individuals. Rather, it may also be relevant to individuals with subclinical autistic traits or other neurodevelopmental or psychiatric conditions, highlighting the potential utility of camouflaging as a transdiagnostic intervention target. Finally, these findings underscore the importance of broader societal change. Public campaigns that challenge stereotypes of autism and promote acceptance of neurodiversity and social difference more broadly may help reduce the stigma that is suggested to contribute to camouflaging in the first place.

## Supplemental Material

sj-docx-1-aut-10.1177_13623613261437500 – Supplemental material for Autistic Traits and Camouflaging: A Meta-Analysis Supplemental material, sj-docx-1-aut-10.1177_13623613261437500 for Autistic Traits and Camouflaging: A Meta-Analysis by Lachlan Greig, Sarah P Coundouris and Julie D Henry in Autism

sj-docx-2-aut-10.1177_13623613261437500 – Supplemental material for Autistic Traits and Camouflaging: A Meta-Analysis Supplemental material, sj-docx-2-aut-10.1177_13623613261437500 for Autistic Traits and Camouflaging: A Meta-Analysis by Lachlan Greig, Sarah P Coundouris and Julie D Henry in Autism

sj-docx-3-aut-10.1177_13623613261437500 – Supplemental material for Autistic Traits and Camouflaging: A Meta-Analysis Supplemental material, sj-docx-3-aut-10.1177_13623613261437500 for Autistic Traits and Camouflaging: A Meta-Analysis by Lachlan Greig, Sarah P Coundouris and Julie D Henry in Autism
