# Supplementary material for: Autistic Traits and Camouflaging: A Meta-Analysis
Source: Autism. 2026 Apr 24;30(6):1398–415. doi: 10.1177/13623613261437500 (PMC13187235; doi:10.1177/13623613261437500)
Supplement: sj-docx-2-aut-10.1177_13623613261437500 – Supplemental material for Autistic Traits and Camouflaging: A Meta-Analysis [file sj-docx-2-aut-10.1177_13623613261437500.docx]

**Supplementary Table 1**

*Study Characteristics and Effect Sizes (R) of Studies Included in the Quantitative Review*

| **Study** | ***N*** | **Gender** | **Diagnosis** | **Age *M*(SD)** | **Other Participant Demographics** | **Depression Measure** | **Anxiety Measure** | **Social Anxiety Measure** | **Autistic Traits Measure** | **Camouflaging Measure** | ***r*** |
| --- | --- | --- | --- | --- | --- | --- | --- | --- | --- | --- | --- |
| Ai et al. (2024) ^a^ | 461 | Male | Non-autistic |  | • USA  • General population;  • Age: 44.2 years overall;  • Current diagnosis: mental health (e.g., anxiety, depression, bipolar) – 12.8%, chronic medical conditions – 9.36%. multi-diagnostic categories – 18.8%. | PHQ-9 | GAD-7 | LSAS | SATQ | CATQ - masking | -0.119 |
|  | 461* | Male | Non-autistic |  |  | PHQ-9 | GAD-7 | LSAS | SATQ | CATQ - compensation | 0.113 |
|  | 461* | Male | Non-autistic |  |  | PHQ-9 | GAD-7 | LSAS | SATQ | CATQ - assimilation | 0.549 |
|  | 491 | Female | Non-autistic |  |  | PHQ-9 | GAD-7 | LSAS | SATQ | CATQ - masking | 0.046 |
|  | 491* | Female | Non-autistic |  |  | PHQ-9 | GAD-7 | LSAS | SATQ | CATQ - compensation | 0.308 |
|  | 491* | Female | Non-autistic |  |  | PHQ-9 | GAD-7 | LSAS | SATQ | CATQ - assimilation | 0.598 |
|  | 20 | Other | Non-autistic |  |  | PHQ-9 | GAD-7 | LSAS | SATQ | CATQ - masking | 0.419 |
|  | 20* | Other | Non-autistic |  |  | PHQ-9 | GAD-7 | LSAS | SATQ | CATQ - compensation | 0.609 |
|  | 20* | Other | Non-autistic |  |  | PHQ-9 | GAD-7 | LSAS | SATQ | CATQ - assimilation | 0.800 |
| Arnold et al. (2023) | 18 |  | Autistic |  | • Australia  • Age at diagnosis: 36.9 years,  • Current diagnosis: anxiety – 72%, depression – 48%, ADHD – 41%, social anxiety – 41%, PTSD – 28%. | PHQ-9 |  |  | AQ-28 | CATQ | 0.21 |
| Atkinson et al. (2025) | 72 |  | Autistic (including self-diagnosed) | 13.38 | • UK  • 83% formally diagnosed with ASD. |  | ASC-ASD |  | AQ-10-A | CATQ | 0.02 |
| Beck et al. (2020) ^a^ | 58 | Female | Mixed | 25.2 (6.17) | • USA  • 18 diagnosed autistic people; all participants self-reported elevated autistic traits e.g., BAPQ > 3);  • Psychiatric diagnosis: any – 63.8%, generalised anxiety – 39.7%, major depressive – 31%, ADHD – 17.2%, social anxiety – 15.5%, learning disorder – 5.2%. | DASS-21 depression | DASS-21 anxiety |  | AQ | CATQ - masking | 0.015 |
|  | 58* | Female | Mixed | 25.2 (6.17) |  | DASS-21 depression | DASS-21 anxiety |  | AQ | CATQ - compensation | 0.516 |
|  | 58* | Female | Mixed | 25.2 (6.17) |  | DASS-21 depression | DASS-21 anxiety |  | AQ | CATQ - assimilation | 0.431 |
|  | 58* | Female | Mixed | 25.2 (6.17) |  | DASS-21 depression | DASS-21 anxiety |  | BAPQ | CATQ - masking | 0.01 |
|  | 58* | Female | Mixed | 25.2 (6.17) |  | DASS-21 depression | DASS-21 anxiety |  | BAPQ | CATQ - compensation | 0.385 |
|  | 58* | Female | Mixed | 25.2 (6.17) |  | DASS-21 depression | DASS-21 anxiety |  | BAPQ | CATQ - assimilation | 0.46 |
|  | 58* | Female | Mixed | 25.2 (6.17) |  | DASS-21 depression | DASS-21 anxiety |  | SRS-2 | CATQ - masking | -0.025 |
|  | 58* | Female | Mixed | 25.2 (6.17) |  | DASS-21 depression | DASS-21 anxiety |  | SRS-2 | CATQ - compensation | 0.491 |
|  | 58* | Female | Mixed | 25.2 (6.17) |  | DASS-21 depression | DASS-21 anxiety |  | SRS-2 | CATQ - assimilation | 0.37 |
|  | 58* | Female | Mixed | 25.2 (6.17) |  | DASS-21 depression | DASS-21 anxiety |  | ADOS-2 Module 4 | CATQ - masking | -0.263 |
|  | 58* | Female | Mixed | 25.2 (6.17) |  | DASS-21 depression | DASS-21 anxiety |  | ADOS-2 Module 4 | CATQ - compensation | 0.247 |
| Beck et al. (2020) ^a^ *continued* | 58* | Female | Mixed | 25.2 (6.17) |  | DASS-21 depression | DASS-21 anxiety |  | ADOS-2 Module 4 | CATQ - assimilation | -0.007 |
| Belcher (2020) | 40 |  | Autistic | 25.65 | • UK  • 20 autistic females, 20 autistic males, 20 non-autistic females, 20 non-autistic males. |  |  |  | AQ | CATQ | 0.249 |
|  | 40 |  | Non-autistic | 27.78 |  |  |  |  | AQ | CATQ | 0.4 |
| Bemmouna (2023) | 89 | Female | Autistic |  | • 588 from France, 2 from Belgium, 4 from Canada, 3 from Switzerland;  • Non-autistic group includes non-clinical and those with BPD • *Autistic participants:* • Psychiatric diagnosis: ADHD – 34%, other psychiatric and / or developmental diagnoses – 71%. | DASS-21 depression | DASS-21 anxiety |  | AQ-S | CATQ | 0.209 |
|  | 46 | Male | Autistic |  |  | DASS-21 depression | DASS-21 anxiety |  | AQ-S | CATQ | 0.268 |
|  | 459 |  | Non-autistic |  |  | DASS-21 depression | DASS-21 anxiety |  | AQ-S | CATQ | 0.529 |
| Black et al. (2019) ^b^ | 7 | Female | Autistic | 34.22 (9.7) | • UK  • Overall IQ: autistic participants – 109.08, non-autistic participants – 102.92. |  |  |  | ADOS-2 Module 4 | AQ discrepancy | -0.936 |
|  | 7* | Female | Autistic | 34.22 (9.7) |  |  |  |  | AQ | ADOS-2 Module 4 discrepancy | 0.56 |
|  | 17 | Male | Autistic | 33.69 (11.4) |  |  |  |  | ADOS-2 Module 4 | AQ discrepancy | -0.84 |
|  | 17* | Male | Autistic | 33.69 (11.4) |  |  |  |  | AQ | ADOS-2 Module 4 discrepancy | 0.67 |
| Bowri (2021) | 237 |  | Autistic | 41.92 (13.3) | • UK  • 139 female, 83 male, 13 other;  • Age at diagnosis: 35.98;  • ASC diagnosis: Asperger’s / HFA – 78.5%, ASD – 17.3%, classic autism – 1.3%, PDD-NOS – 3%;  • Co-occurring diagnoses: anxiety – 56.5%, depression – 54%, ADHD – 10.5%, ID / LD – 4.6%, other – 33.3%. | PHQ-9 | GAD-7 | LSAS | BAPQ | CATQ | 0.257 |
| Bradley et al. (2025) | 180 |  | Autistic (including self-diagnosed) | 38 (13.6) | • UK  • Diagnosis: clinical – 80.6%, self-diagnosis – 19.4%;  • No participants reported an intellectual disability;  • Eating disorder type: multiple – 35.3%, anorexia nervosa – 33.8%, bulimia nervosa – 9.2%, binge disorder – 7.7%, other – 13.9%;  • Other mental health diagnosis: yes – 64.4%. |  |  |  | AQ-10 | CATQ | 0.264 |
| Cardon et al. (2023) ^a^ | 556 | Female | Non-autistic | 21.21 (1.92) | • USA  • All university students | DASS-21 depression | DASS-21 anxiety |  | BAPQ | CATQ - masking | 0.13 |
|  | 556* | Female | Non-autistic | 21.21 (1.92) |  | DASS-21 depression | DASS-21 anxiety |  | BAPQ | CATQ - compensation | 0.535 |
|  | 556* | Female | Non-autistic | 21.21 (1.92) |  | DASS-21 depression | DASS-21 anxiety |  | BAPQ | CATQ - assimilation | 0.063 |
|  | 556* | Female | Non-autistic | 21.21 (1.92) |  | DASS-21 depression | DASS-21 anxiety |  | AQ | CATQ - masking | 0.135 |
|  | 556* | Female | Non-autistic | 21.21 (1.92) |  | DASS-21 depression | DASS-21 anxiety |  | AQ | CATQ - compensation | 0.529 |
|  | 556* | Female | Non-autistic | 21.21 (1.92) |  | DASS-21 depression | DASS-21 anxiety |  | AQ | CATQ - assimilation | 0.087 |
|  | 558 | Male | Non-autistic | 22.2 (1.75) |  | DASS-21 depression | DASS-21 anxiety |  | BAPQ | CATQ - masking | 0.073 |
|  | 558* | Male | Non-autistic | 22.2 (1.75) |  | DASS-21 depression | DASS-21 anxiety |  | BAPQ | CATQ - compensation | 0.446 |
| Cardon et al. (2023) ^a^ *continued* | 558* | Male | Non-autistic | 22.2 (1.75) |  | DASS-21 depression | DASS-21 anxiety |  | BAPQ | CATQ - assimilation | -0.131 |
|  | 558* | Male | Non-autistic | 22.2 (1.75) |  | DASS-21 depression | DASS-21 anxiety |  | AQ | CATQ - masking | 0.061 |
|  | 558* | Male | Non-autistic | 22.2 (1.75) |  | DASS-21 depression | DASS-21 anxiety |  | AQ | CATQ -compensation | 0.395 |
|  | 558* | Male | Non-autistic | 22.2 (1.75) |  | DASS-21 depression | DASS-21 anxiety |  | AQ | CATQ - assimilation | -0.068 |
| Cassidy et al. (2020) | 160 |  | Non-autistic |  | • UK  • 139 female, 21 male;  • All university students. | PHQ-9 | GAD-7 |  | AQ-S | CATQ - masking | 0.309 |
|  | 160* |  | Non-autistic |  |  | PHQ-9 | GAD-7 |  | AQ-S | CATQ - compensation | 0.428 |
|  | 160* |  | Non-autistic |  |  | PHQ-9 | GAD-7 |  | AQ-S | CATQ – assimilation | 0.589 |
| Cassidy et al. (2018) | 164 |  | Autistic | 40.21 | • UK  • 99 female, 65 male;  • Age at diagnosis: 34.81 years;  • ASC subtype: AS / HFA – 82.9%, classic autism – 1.2%, ASC – 8.5%, PDD-NOS – 1.2%, other – 6%;  • Support needs: need / receive support – 76.8%, unmet needs – 3.7%;  • Mental health condition: depression – 79.9%, anxiety – 71.3%, OCD – 14.6%, personality disorder – 14%;  • Developmental condition: dyspraxia – 11%, learning disability – 0.6%, dyslexia – 7.9%, ADHD – 6.7%, developmental delay – 0.6%. |  |  |  | AQ | CATQ | 0.058 |
| Cassidy et al. (2021) | 268 |  | Non-autistic | 41.57 (14.1) | • UK  • 82 autistic male, 226 autistic female, 29 possibly autistic male, 84 possibly autistic female, 85 non-autistic male, 183 non-autistic female;  • *Autistic and possibly autistic participants:*  • Identify with a different gender than birth sex – 16.15%;  • Developmental conditions: dyspraxia – 6.65%, learning disability – 1.19%, dyscalculia – 1.43%, dyslexia – 6.41%, ADHD – 12.11%, developmental delay – 1.19%;  • Current mental health conditions: depression – 47.27%, anxiety – 55.58%, OCD – 7.6%, bipolar –4.04%, personality disorder – 6.89%, PTSD – 16.63%, anorexia – 3.09%, bulimia – 1.43%, Tourette’s – 1.43%, other – 8.31%. | PHQ-9 | ASA-A |  | AQ-S | CATQ | 0.659 |
|  | 421 |  | Mixed |  |  | PHQ-9 | ASA-A |  | AQ-S | CATQ | 0.233 |
| Cassidy et al. (2023) | 180 |  | Mixed | 21.09 (6) | • UK  • 138 female, 35 male, 7 other; • Current diagnoses: autism – 1.7%, suspected autism – 8.3%, non-autistic – 89.4%, depression – 19.4%, anxiety – 22.2%, OCD – 3.3%. |  |  |  | AQ-10 | CATQ | 0.491 |
| Coburn (2022) ^a^ | 7 | Female | Autistic (including self-diagnosed) | 36 (10) | • USA  • 6 cis woman, 1 trans woman, 4 nonbinary, 1 autigender, 3 trans man, 5 cis man; • Age of diagnosis: 22.3 years;  • Diagnosis: formal – 80%, self-diagnosed – 20%. | DASS-21 depression | DASS-21 anxiety |  | RAADS-14 | CATQ - masking | 0.92 |
|  | 7* | Female | Autistic (including self-diagnosed) | 36 (10) |  | DASS-21 depression | DASS-21 anxiety |  | RAADS-14 | CATQ - compensation | 0.722 |
| Coburn (2022) ^a^ *continued* | 7* | Female | Autistic (including self-diagnosed) | 36 (10) |  | DASS-21 depression | DASS-21 anxiety |  | RAADS-14 | CATQ - assimilation | 0.81 |
|  | 8 | Male | Autistic (including self-diagnosed) | 33.13 (10.8) |  | DASS-21 depression | DASS-21 anxiety |  | RAADS-14 | CATQ - masking | 0.391 |
|  | 8* | Male | Autistic (including self-diagnosed) | 33.13 (10.8) |  | DASS-21 depression | DASS-21 anxiety |  | RAADS-14 | CATQ - compensation | 0.642 |
|  | 8* | Male | Autistic (including self-diagnosed) | 33.13 (10.8) |  | DASS-21 depression | DASS-21 anxiety |  | RAADS-14 | CATQ - assimilation | 0.485 |
|  | 5 | Other | Autistic (including self-diagnosed) | 28 (9.03) |  | DASS-21 depression | DASS-21 anxiety |  | RAADS-14 | CATQ - masking | -0.962 |
|  | 5* | Other | Autistic (including self-diagnosed) | 28 (9.03) |  | DASS-21 depression | DASS-21 anxiety |  | RAADS-14 | CATQ - compensation | -0.856 |
|  | 5* | Other | Autistic (including self-diagnosed) | 28 (9.03) |  | DASS-21 depression | DASS-21 anxiety |  | RAADS-14 | CATQ - assimilation | -0.531 |
| Dell'Osso et al. (2022) | 2439 |  | Non-autistic | 26.92 | • Italy  • 1415 female, 726 male; |  |  |  | AdAS Spectrum | CATQ | 0.652 |
| Dodd (2022) | 201 |  | Autistic (including self-diagnosed) | 36.63 (13.2) | • UK  • 100 autistic participants (50 female, 37 male, 11 other, 2 did not disclose) and 101 self-diagnosed autistic participants (50 female, 22, male, 28 other, 1 did not disclose); • Age of formal diagnosis: 30.38 years. | BDI-II | BAI |  | AQ | CATQ | 0.06 |
| Dodd (2022) *continued* | 208 |  | Autistic (including self-diagnosed) | 35.39 (11.3) | • 208 diagnosed and self-diagnosed autistic participants (149 female, 36 male, 23 other); • Age of diagnosis: 30.04 years, time elapsed since diagnosis: 5.38 years; • Formal diagnosis: 65.4%. | BDI-II | BAI |  | AQ | CATQ | 0.18 |
| English et al. (2021) ^b^ | 94 | Male | Non-autistic | 33.34 (11.4) | • Australia |  |  |  | AQ (SOC+COM) | CATQ - masking | 0.171 |
|  | 94* | Male | Non-autistic | 33.34 (11.4) |  |  |  |  | AQ (SOC+COM) | CATQ - compensation | 0.314 |
|  | 94* | Male | Non-autistic | 33.34 (11.4) |  |  |  |  | AQ (SOC+COM) | CATQ - assimilation | 0.799 |
|  | 94* | Male | Non-autistic | 33.34 (11.4) |  |  |  |  | CATI | CATQ - masking | 0.453 |
|  | 94* | Male | Non-autistic | 33.34 (11.4) |  |  |  |  | CATI | CATQ - compensation | 0.689 |
|  | 94* | Male | Non-autistic | 33.34 (11.4) |  |  |  |  | CATI | CATQ - assimilation | 0.703 |
|  | 86 | Female | Non-autistic | 35.21 (12.3) |  |  |  |  | AQ (SOC+COM) | CATQ - masking | -0.037 |
|  | 86* | Female | Non-autistic | 35.21 (12.3) |  |  |  |  | AQ (SOC+COM) | CATQ - compensation | 0.407 |
|  | 86* | Female | Non-autistic | 35.21 (12.3) |  |  |  |  | AQ (SOC+COM) | CATQ - assimilation | 0.795 |
|  | 86* | Female | Non-autistic | 35.21 (12.3) |  |  |  |  | CATI | CATQ - masking | 0.223 |
| English et al. (2021) ^b^ *continued* | 86* | Female | Non-autistic | 35.21 (12.3) |  |  |  |  | CATI | CATQ - compensation | 0.653 |
|  | 86* | Female | Non-autistic | 35.21 (12.3) |  |  |  |  | CATI | CATQ - assimilation | 0.801 |
|  | 6 | Other | Non-autistic | 27.33 (6.98) |  |  |  |  | AQ (SOC+COM) | CATQ - masking | -0.197 |
|  | 6* | Other | Non-autistic | 27.33 (6.98) |  |  |  |  | AQ (SOC+COM) | CATQ - compensation | 0.478 |
|  | 6* | Other | Non-autistic | 27.33 (6.98) |  |  |  |  | AQ (SOC+COM) | CATQ - assimilation | 0.728 |
|  | 6* | Other | Non-autistic | 27.33 (6.98) |  |  |  |  | CATI | CATQ - masking | 0.547 |
|  | 6* | Other | Non-autistic | 27.33 (6.98) |  |  |  |  | CATI | CATQ - compensation | 0.771 |
|  | 6* | Other | Non-autistic | 27.33 (6.98) |  |  |  |  | CATI | CATQ - assimilation | 0.842 |
| Funawatari et al. (2024) ^a^ | 48 | Female | Autistic | 33.50 (9.8) | • Japan  • Comorbidities: ADHD – 23%, SLD – 4.8%, IDD – 5.8%. |  |  |  | AQ | Modified CC | 0.173 |
|  | 56 | Male | Autistic | 38.40 (11.1) |  |  |  |  | AQ | Modified CC | -0.103 |
| Galvin et al. (2024) ^a^ | 142 | Male | Autistic | 29.72 (11.5) | • UK  • Other diagnoses: anxiety – 52%, depression – 54%, OCD – 12%, ADHD – 18%. | PHQ-9 | GAD-7 | LSAS | AQ | CATQ - masking | 0.065 |
|  | 142* | Male | Autistic | 29.72 (11.5) |  | PHQ-9 | GAD-7 | LSAS | AQ | CATQ - compensation | 0.494 |
|  | 142* | Male | Autistic | 29.72 (11.5) |  | PHQ-9 | GAD-7 | LSAS | AQ | CATQ - assimilation | 0.704 |
| Galvin et al. (2024) ^a^ *continued* | 152 | Female | Autistic | 31.28 (13.5) |  | PHQ-9 | GAD-7 | LSAS | AQ | CATQ - masking | 0.397 |
|  | 152* | Female | Autistic | 31.28 (13.5) |  | PHQ-9 | GAD-7 | LSAS | AQ | CATQ - compensation | 0.739 |
|  | 152* | Female | Autistic | 31.28 (13.5) |  | PHQ-9 | GAD-7 | LSAS | AQ | CATQ - assimilation | 0.803 |
| Hannon et al. (2023) ^b^ | 21 | Female | Autistic |  | • UK  • IQ: 100.85. |  |  |  | AQ | CATQ - masking | -0.023 |
|  | 21* | Female | Autistic |  |  |  |  |  | AQ | CATQ - compensation | 0.237 |
|  | 21* | Female | Autistic |  |  |  |  |  | AQ | CATQ - assimilation | 0.209 |
|  | 21* | Female | Autistic |  |  |  |  |  | ADOS-2 Module 3&4 | CATQ - masking | -0.263 |
|  | 21* | Female | Autistic |  |  |  |  |  | ADOS-2 Module 3&4 | CATQ - compensation | 0.173 |
|  | 21* | Female | Autistic |  |  |  |  |  | ADOS-2 Module 3&4 | CATQ - assimilation | 0.109 |
|  | 21* | Female | Autistic |  |  |  |  |  | ADOS-2 Module 3&4 | AQ discrepancy | 0.946 |
|  | 21* | Female | Autistic |  |  |  |  |  | AQ | ADOS-2 Module 4 discrepancy | 0.095 |
|  | 22 | Male | Autistic |  |  |  |  |  | AQ | CATQ - masking | 0.145 |
|  | 22* | Male | Autistic |  |  |  |  |  | AQ | CATQ - compensation | 0.201 |
| Hannon et al. (2023) ^b^ *continued* | 22* | Male | Autistic |  |  |  |  |  | AQ | CATQ - assimilation | 0.453 |
|  | 22* | Male | Autistic |  |  |  |  |  | ADOS-2 Module 3&4 | CATQ - masking | -0.088 |
|  | 22* | Male | Autistic |  |  |  |  |  | ADOS-2 Module 3&4 | CATQ - compensation | -0.158 |
|  | 22* | Male | Autistic |  |  |  |  |  | ADOS-2 Module 3&4 | CATQ - assimilation | 0.138 |
|  | 22* | Male | Autistic |  |  |  |  |  | ADOS-2 Module 3&4 | AQ discrepancy | 0.934 |
|  | 22* | Male | Autistic |  |  |  |  |  | AQ | ADOS-2 Module 4 discrepancy | -0.153 |
| Hartman et al. (2023) ^a^ | 10 | Male | Autistic | 35.6 (9.14) | • Canada |  |  |  | AQ | CATQ | -0.008 |
|  | 23 | Female | Autistic | 36.35 (9.62) |  |  |  |  | AQ | CATQ | 0.303 |
|  | 18 | Male | Non-autistic | 21.39 (6.71) |  |  |  |  | AQ | CATQ | -0.25 |
|  | 16 | Female | Non-autistic | 23.81 (10.1) |  |  |  |  | AQ | CATQ | 0.281 |
| Howard & Sedgewick (2021) | 245 |  | Autistic (including self-diagnosed) |  | • UK  • 245 autistic participants (151 female, 61 male, 31 non-binary, 2 did not respond, 5 transgender); • Age: 40.44 years, age of diagnosis: 34.38 years; • Diagnosis: ASD – 44.9%, AS – 37.5%, PDD-NOS – 0.82%, self-diagnosed – 16.3%. |  | GAD-7 |  | AQ-10 | CATQ - masking | 0.088 |
| Howard & Sedgewick (2021) continued | 245* |  | Autistic (including self-diagnosed) |  |  |  | GAD-7 |  | AQ-10 | CATQ - compensation | 0.018 |
|  | 245* |  | Autistic (including self-diagnosed) |  |  |  | GAD-7 |  | AQ-10 | CATQ - assimilation | -0.02 |
| Hull et al. (2021) ^b^ | 177 | Female | Autistic | 39.7 (12.8) | • UK  • Diagnosis: GAD – 56.7%, depression – 54.4%, SAD / social phobia – 2.3%, 1 additional diagnosis – 25%, 2 additional diagnoses – 14%, 3 or more additional diagnoses – 9%. |  |  | LSAS | BAPQ | CATQ - masking | 0.078 |
|  | 177* | Female | Autistic | 39.7 (12.8) |  |  |  | LSAS | BAPQ | CATQ - compensation | 0.141 |
|  | 177* | Female | Autistic | 39.7 (12.8) |  |  |  | LSAS | BAPQ | CATQ - assimilation | 0.059 |
|  | 99 | Male | Autistic | 46.48 (14) |  |  |  | LSAS | BAPQ | CATQ - masking | 0.134 |
|  | 99* | Male | Autistic | 46.48 (14) |  |  |  | LSAS | BAPQ | CATQ - compensation | 0.096 |
|  | 99* | Male | Autistic | 46.48 (14) |  |  |  | LSAS | BAPQ | CATQ - assimilation | -0.038 |
|  | 19 | Other | Autistic | 33.74 (11.2) |  |  |  | LSAS | BAPQ | CATQ - masking | 0.231 |
|  | 19* | Other | Autistic | 33.74 (11.2) |  |  |  | LSAS | BAPQ | CATQ - compensation | 0.078 |
|  | 19* | Other | Autistic | 33.74 (11.2) |  |  |  | LSAS | BAPQ | CATQ - assimilation | -0.292 |
| Keating et al. (2024) | 131 | Female | Autistic | 30.94 (9.01) | • Mixed countries | DASS-21 depression | DASS-21 anxiety |  | AQ | CATQ | 0.442 |
|  | 137 | Male | Autistic | 30.19 (9.31) |  | DASS-21 depression | DASS-21 anxiety |  | AQ | CATQ | 0.372 |
| Keating et al. (2024) *continued* | 40 | Other | Autistic | 28.68 (8.57) |  | DASS-21 depression | DASS-21 anxiety |  | AQ | CATQ | 0.358 |
| Kuo et al. (2024) ^a^ | 146 | Female | Mixed | 24.11 (4.7) | • Taiwan  • History of diagnosed disorder: anxiety – 19.7%, depressive – 21.3%, bipolar – 3.8%, ASD – 2.7%. | PHQ-9 | GAD-7 |  | AQ-35 | CATQ – masking / compensation | 0.467 |
|  | 146* | Female | Mixed | 24.11 (4.7) |  | PHQ-9 | GAD-7 |  | AQ-35 | CATQ - assimilation | 0.627 |
|  | 37 | Male | Mixed | 23 (4.48) |  | PHQ-9 | GAD-7 |  | AQ-35 | CATQ – masking / compensation | 0.112 |
|  | 37* | Male | Mixed | 23 (4.48) |  | PHQ-9 | GAD-7 |  | AQ-35 | CATQ - assimilation | 0.579 |
| Lei et al. (2024) ^a^ | 17 | Male | Autistic | 16.06 (1.6) | • UK  • Co-occurring diagnosis: any mental health condition – 80.33%, any physical health condition – 27.87%, any co-occurring – 88.52%; • Mental health condition: ADHD – 14.75%, GAD – 36.07%, SAD – 31.15%, OCD – 34.43%, panic – 1.64%, PTSD – 3.28%, depression – 27.87%, ED – 9.84%; | RCADS-dep | RCADS-GAD |  | AQ-28 | CATQ - masking | 0.057 |
|  | 17* | Male | Autistic | 16.06 (1.6) |  | RCADS-dep | RCADS-GAD |  | AQ-28 | CATQ - compensation | 0.28 |
|  | 17* | Male | Autistic | 16.06 (1.6) |  | RCADS-dep | RCADS-GAD |  | AQ-28 | CATQ - assimilation | 0.638 |
|  | 36 | Female | Autistic | 16.47 (1.77) |  | RCADS-dep | RCADS-GAD |  | AQ-28 | CATQ - masking | 0.033 |
|  | 36* | Female | Autistic | 16.47 (1.77) |  | RCADS-dep | RCADS-GAD |  | AQ-28 | CATQ - compensation | 0.229 |
|  | 36* | Female | Autistic | 16.47 (1.77) |  | RCADS-dep | RCADS-GAD |  | AQ-28 | CATQ - assimilation | 0.365 |
| Lei et al. (2024) ^a^ *continued* | 8 | Other | Autistic | 16.38 (1.69) |  | RCADS-dep | RCADS-GAD |  | AQ-28 | CATQ - masking | 0.642 |
|  | 8* | Other | Autistic | 16.38 (1.69) |  | RCADS-dep | RCADS-GAD |  | AQ-28 | CATQ - compensation | 0.784 |
|  | 8* | Other | Autistic | 16.38 (1.69) |  | RCADS-dep | RCADS-GAD |  | AQ-28 | CATQ - assimilation | 0.284 |
|  | 11 | Male | Non-autistic | 15.91 (1.84) | • Co-occurring diagnosis: any mental health condition – 70.37%, any physical health condition – 14.81%, any co-occurring – 72.22%; • Mental health condition: ADHD – 0%, GAD – 35.19, SAD – 14.81%, OCD – 27.78, panic – 1.85%, PTSD – 7.41%, depression – 24.07%, ED – 5.56%. | RCADS-dep | RCADS-GAD |  | AQ-28 | CATQ - masking | -0.26 |
|  | 11* | Male | Non-autistic | 15.91 (1.84) |  | RCADS-dep | RCADS-GAD |  | AQ-28 | CATQ - compensation | 0.268 |
|  | 11* | Male | Non-autistic | 15.91 (1.84) |  | RCADS-dep | RCADS-GAD |  | AQ-28 | CATQ - assimilation | 0.608 |
|  | 37 | Female | Non-autistic | 16.03 (1.54) |  | RCADS-dep | RCADS-GAD |  | AQ-28 | CATQ - masking | 0.411 |
|  | 37* | Female | Non-autistic | 16.03 (1.54) |  | RCADS-dep | RCADS-GAD |  | AQ-28 | CATQ - compensation | 0.554 |
|  | 37* | Female | Non-autistic | 16.03 (1.54) |  | RCADS-dep | RCADS-GAD |  | AQ-28 | CATQ - assimilation | 0.745 |
|  | 4 | Other | Non-autistic | 17 (0.82) |  | RCADS-dep | RCADS-GAD |  | AQ-28 | CATQ - masking | 0.684 |
|  | 4* | Other | Non-autistic | 17 (0.82) |  | RCADS-dep | RCADS-GAD |  | AQ-28 | CATQ - compensation | 0.785 |
|  | 4* | Other | Non-autistic | 17 (0.82) |  | RCADS-dep | RCADS-GAD |  | AQ-28 | CATQ – assimilation | 0.784 |
| Livingston et al. (2020) | 117 |  | Mixed | 34.85 (13.3) | • UK  • 14 autistic male, 44 autistic female, 8 non-autistic male, 51 non-autistic female; • Age of diagnosis: 30.14 years. |  |  |  | AQ-10 | CC - masking | 0.07 |
|  | 117* |  | Mixed | 34.85 (13.3) |  |  |  |  | AQ-10 | CC - compensation | 0.26 |
|  | 117* |  | Mixed | 34.85 (13.3) |  |  |  |  | AQ-10 | CC - accomodation | 0.05 |
| Lu et al. (2023) | 1215 |  | Non-autistic |  | • China  • Diagnosis: with MDS – 28.72%, without MDS – 71.28%; • Elevated autistic traits: yes – 4.69%, no – 95.31%. | PHQ-9 |  |  | AQ | CATQ | 0.218 |
| Lui et al. (2023) ^a^ | 61 | Female | Mixed | 20.67 (1.03) | • China  • 94 participants (6 diagnosed autistic people); • University students | CES-D | GAD-7 |  | ADOS-2 | AQ discrepancy | 0.136 |
|  | 61* | Female | Mixed | 20.67 (1.03) |  | CES-D | GAD-7 |  | AQ | ADOS-2 Discrepancy | 0.989 |
|  | 33 | Male | Mixed | 21.06 (1.27) |  | CES-D | GAD-7 |  | ADOS-2 | AQ discrepancy | 0.121 |
|  | 33* | Male | Mixed | 21.06 (1.27) |  | CES-D | GAD-7 |  | AQ | ADOS-2 Discrepancy | 0.969 |
| Lundin Remnelius & Bolte (2023) | 523 |  | Mixed | 36.89 | • Sweden  • Autistic participants: • Co-occurring condition: any – 50%, ADHD – 41%, IDD – 5%, other neurodevelopmental conditions – 7%; • Non-autistic participants: • Psychiatric condition: any – 21%, ADHD – 9%, IDD – 0.6%, other neurodevelopmental conditions – 6%. |  |  |  | AQ | CATQ - masking | 0.25 |
| Lundin Remnelius & Bolte (2023) *continued* | 523* |  | Mixed | 36.89 |  |  |  |  | AQ | CATQ - compensation | 0.38 |
|  | 523* |  | Mixed | 36.89 |  |  |  |  | AQ | CATQ - assimilation | 0.53 |
|  | 116 |  | Mixed | 11.8 |  |  |  |  | AQ | CATQ - masking | 0.09 |
|  | 116* |  | Mixed | 11.8 |  |  |  |  | AQ | CATQ - compensation | 0.17 |
|  | 116* |  | Mixed | 11.8 |  |  |  |  | AQ | CATQ - assimilation | 0.48 |
| McKinnon et al. (2025) | 308 |  | Autistic (including self-diagnosed) | 36.17 (11.62) | • Australia  • Diagnosis: formal – 86%, self-diagnosed – 14%;  • Gender: female – 61.4%, male – 28.6%, non-binary / other genders / undisclosed – 10.1%;  • Mental health diagnosis: anxiety – 55.52%, depression – 51.62%, PTSD – 9.42%, OCD – 6.82%, ED – 1.62%;  • Number of co-occuring mental health diagnoses: 0 – 30.84%, 1 – 23.05%, 2 – 33.12%, 3+ - 12.99%. |  |  | SIAS / SPS | BAPQ | CATQ - masking | 0 |
|  | 308 |  | Autistic (including self-diagnosed) | 36.17 (11.62) |  |  |  | SIAS / SPS | BAPQ | CATQ - compensation | 0.24 |
|  | 308 |  | Autistic (including self-diagnosed) | 36.17 (11.62) |  |  |  | SIAS / SPS | BAPQ | CATQ - assimilation | 0.66 |
| Milner et al. (2023) | 34 | Male | Autistic | 22.52 | • UK |  |  |  | SRS-2 | CATQ | 0.208 |
|  | 42 | Female | Autistic | 22.58 |  |  |  |  | SRS-2 | CATQ | 0.377 |
|  | 89 | Male | Non-autistic | 22.49 |  |  |  |  | SRS-2 | CATQ | 0.369 |
| Milner et al. (2023) *continued* | 87 | Female | Non-autistic | 22.42 |  |  |  |  | SRS-2 | CATQ | 0.481 |
|  | 40 | Male | Non-autistic | 22.14 |  |  |  |  | SRS-2 | CATQ | 0.265 |
|  | 90 | Female | Non-autistic | 22.34 |  |  |  |  | SRS-2 | CATQ | 0.433 |
| Moore et al. (2023) | 512 |  | Mixed |  | • UK  • Diagnosis: formal – 59.6%, suspected, pursuing diagnosis – 20.1%, suspected, not pursuing diagnosis – 20.3%; • Diagnosed mental health condition: yes – 77.2%, no – 21.5%, prefer not to say – 1.3%; • Mental health condition: anxiety – 60.1%, bipolar – 4.8%, depressive disorder – 58.9%, OCD – 12.9%, schizophrenic disorders – 0.8%, ED – 13.7%, personality disorders – 6.9%, trauma or stress disorders – 24.6%, other – 13.6%. |  |  |  | AQ-10 | CATQ - masking | -0.071 |
|  | 512* |  | Mixed |  |  |  |  |  | AQ-10 | CATQ - compensation | 0.217 |
|  | 512* |  | Mixed |  |  |  |  |  | AQ-10 | CATQ - assimilation | 0.306 |
| Nieradka & Kossewska (2023) ^a^ | 68 | Female | Non-autistic | 24.3 (4.58) | • Poland |  |  |  | AQ-10 | CATQ - masking | 0.359 |
|  | 68* | Female | Non-autistic | 24.3 (4.58) |  |  |  |  | AQ-10 | CATQ - compensation | 0.665 |
|  | 68* | Female | Non-autistic | 24.3 (4.58) |  |  |  |  | AQ-10 | CATQ - assimilation | 0.678 |
|  | 36 | Male | Non-autistic | 25.4 (4.53) |  |  |  |  | AQ-10 | CATQ - masking | -0.198 |
|  | 36* | Male | Non-autistic | 25.4 (4.53) |  |  |  |  | AQ-10 | CATQ - compensation | 0.212 |
| Nieradka & Kossewska ^a^ (2023) *continued* | 36* | Male | Non-autistic | 25.4 (4.53) |  |  |  |  | AQ-10 | CATQ - assimilation | 0.573 |
| Okada et al. (2024) ^b^ | 170 | Male | Non-autistic |  | • Japan  • All university students;  • Longitudinal design. |  |  |  | CATI-J | CATQ - masking | -0.025 |
|  | 170* | Male | Non-autistic |  |  |  |  |  | CATI-J | CATQ - compensation | 0.352 |
|  | 170* | Male | Non-autistic |  |  |  |  |  | CATI-J | CATQ - assimilation | 0.682 |
|  | 326 | Female | Non-autistic |  |  |  |  |  | CATI-J | CATQ - masking | 0.300 |
|  | 326* | Female | Non-autistic |  |  |  |  |  | CATI-J | CATQ - compensation | 0.676 |
|  | 326* | Female | Non-autistic |  |  |  |  |  | CATI-J | CATQ - assimilation | 0.754 |
|  | 13 | Other | Non-autistic |  |  |  |  |  | CATI-J | CATQ - masking | -0.230 |
|  | 13* | Other | Non-autistic |  |  |  |  |  | CATI-J | CATQ - compensation | 0.430 |
|  | 13* | Other | Non-autistic |  |  |  |  |  | CATI-J | CATQ - assimilation | -0.204 |
| O'Loghlen et al. (2024) | 278 |  | Non-autistic | 25.02 (8.87) | • Australia |  |  |  | BAPQ | CATQ - masking | 0.24 |
|  | 278* |  | Non-autistic | 25.02 (8.87) |  |  |  |  | BAPQ | CATQ - compensation | 0.52 |
| O'Loghlen et al. (2024) *continued* | 278* |  | Non-autistic | 25.02 (8.87) |  |  |  |  | BAPQ | CATQ - assimilation | 0.72 |
| Oshima et al. (2024) ^b^ | 78 | Female | Autistic | 35.97 | • Japan  • Diagnosis: ASD – 62%, developmental disability – 26%, PDD – 35%, autistic disorder – 18%, Asperger disorder – 35%, ADHD – 28%. | PHQ-9 | GAD-7 | LSAS | BAPQ | CATQ - masking | -0.047 |
|  | 78* | Female | Autistic | 35.97 |  | PHQ-9 | GAD-7 | LSAS | BAPQ | CATQ - compensation | 0.039 |
|  | 78* | Female | Autistic | 35.97 |  | PHQ-9 | GAD-7 | LSAS | BAPQ | CATQ - assimilation | 0.623 |
|  | 113 | Male | Autistic | 39.44 |  | PHQ-9 | GAD-7 | LSAS | BAPQ | CATQ - masking | 0.009 |
|  | 113* | Male | Autistic | 39.44 |  | PHQ-9 | GAD-7 | LSAS | BAPQ | CATQ - compensation | 0.131 |
|  | 113* | Male | Autistic | 39.44 |  | PHQ-9 | GAD-7 | LSAS | BAPQ | CATQ – assimilation | 0.697 |
| Perry et al. (2022) | 223 |  |  | 34.19 (11) | • UK  • Age of diagnosis: 28.67 years. |  |  |  | RAADS-14 | CATQ | 0.19 |
| Porricelli et al. (2024) ^a^ | 48 | Male | Non-autistic | 28.25 (6.44) | • UK | PHQ-8 |  |  | AQ-10 | CATQ - masking | 0.359 |
|  | 48* | Male | Non-autistic | 28.25 (6.44) |  | PHQ-8 |  |  | AQ-10 | CATQ - compensation | 0.573 |
|  | 48* | Male | Non-autistic | 28.25 (6.44) |  | PHQ-8 |  |  | AQ-10 | CATQ – assimilation | 0.446 |
|  | 120 | Female | Non-autistic | 31.33 (10.99) |  | PHQ-8 |  |  | AQ-10 | CATQ - masking | 0.223 |
|  | 120* | Female | Non-autistic | 31.33 (10.99) |  | PHQ-8 |  |  | AQ-10 | CATQ - compensation | 0.570 |
|  | 120* | Female | Non-autistic | 31.33 (10.99) |  | PHQ-8 |  |  | AQ-10 | CATQ – assimilation | 0.579 |
| Pyszkowska (2024) ^b^ | 114 | Female | Autistic | 29.87 | • Poland  • Participants were either diagnosed with ASD, SAD, or ASD & SAD;  • No participants had an intellectual disability; |  |  | LSAS-SR | AQ-10 | CATQ - masking | 0.058 |
|  | 114* | Female | Autistic | 29.87 |  |  |  | LSAS-SR | AQ-10 | CATQ - compensation | 0.343 |
|  | 114* | Female | Autistic | 29.87 |  |  |  | LSAS-SR | AQ-10 | CATQ – assimilation | 0.357 |
|  | 13 | Male | Autistic | 25.23 |  |  |  | LSAS-SR | AQ-10 | CATQ - masking | -0.151 |
|  | 13* | Male | Autistic | 25.23 |  |  |  | LSAS-SR | AQ-10 | CATQ - compensation | 0.271 |
|  | 13* | Male | Autistic | 25.23 |  |  |  | LSAS-SR | AQ-10 | CATQ – assimilation | 0.420 |
|  | 23 | Other | Autistic | 27.13 |  |  |  | LSAS-SR | AQ-10 | CATQ - masking | -0.229 |
|  | 23* | Other | Autistic | 27.13 |  |  |  | LSAS-SR | AQ-10 | CATQ - compensation | 0.015 |
|  | 23* | Other | Autistic | 27.13 |  |  |  | LSAS-SR | AQ-10 | CATQ – assimilation | 0.260 |
|  | 50 | Female | Non-autistic | 27.48 |  |  |  | LSAS-SR | AQ-10 | CATQ - masking | 0.017 |
|  | 50* | Female | Non-autistic | 27.48 |  |  |  | LSAS-SR | AQ-10 | CATQ - compensation | 0.011 |
|  | 50* | Female | Non-autistic | 27.48 |  |  |  | LSAS-SR | AQ-10 | CATQ – assimilation | 0.159 |
|  | 7 | Male | Non-autistic | 29.57 |  |  |  | LSAS-SR | AQ-10 | CATQ - masking | 0.539 |
|  | 7* | Male | Non-autistic | 29.57 |  |  |  | LSAS-SR | AQ-10 | CATQ - compensation | 0.759 |
| Pyszkowska (2024) ^b^ *continued* | 7* | Male | Non-autistic | 29.57 |  |  |  | LSAS-SR | AQ-10 | CATQ – assimilation | 0.785 |
|  | 8 | Other | Non-autistic | 24.75 |  |  |  | LSAS-SR | AQ-10 | CATQ - masking | 0.172 |
|  | 8* | Other | Non-autistic | 24.75 |  |  |  | LSAS-SR | AQ-10 | CATQ - compensation | 0.480 |
|  | 8* | Other | Non-autistic | 24.75 |  |  |  | LSAS-SR | AQ-10 | CATQ – assimilation | 0.777 |
|  | 26 | Female | Autistic | 29.42 |  |  |  | LSAS-SR | AQ-10 | CATQ - masking | 0.009 |
|  | 26* | Female | Autistic | 29.42 |  |  |  | LSAS-SR | AQ-10 | CATQ - compensation | -0.010 |
|  | 26* | Female | Autistic | 29.42 |  |  |  | LSAS-SR | AQ-10 | CATQ – assimilation | 0.412 |
|  | 9 | Male | Autistic | 27.56 |  |  |  | LSAS-SR | AQ-10 | CATQ - masking | 0.013 |
|  | 9* | Male | Autistic | 27.56 |  |  |  | LSAS-SR | AQ-10 | CATQ - compensation | 0.504 |
|  | 9* | Male | Autistic | 27.56 |  |  |  | LSAS-SR | AQ-10 | CATQ – assimilation | 0.214 |
|  | 10 | Other | Autistic | 26 |  |  |  | LSAS-SR | AQ-10 | CATQ - masking | -0.301 |
|  | 10* | Other | Autistic | 26 |  |  |  | LSAS-SR | AQ-10 | CATQ - compensation | 0.098 |
|  | 10* | Other | Autistic | 26 |  |  |  | LSAS-SR | AQ-10 | CATQ – assimilation | 0.374 |
| Robinson et al. (2020) | 278 |  | Autistic |  | • UK |  |  |  | BAPQ | CATQ - masking | -0.022 |
|  | 278* |  | Autistic |  |  |  |  |  | BAPQ | CATQ - compensation | 0.19 |
| Robinson et al. (2020) *continued* | 278* |  | Autistic |  |  |  |  |  | BAPQ | CATQ - assimilation | 0.651 |
|  | 230 |  | Non-autistic |  |  |  |  |  | BAPQ | CATQ - masking | 0.271 |
|  | 230* |  | Non-autistic |  |  |  |  |  | BAPQ | CATQ - compensation | 0.645 |
|  | 230* |  | Non-autistic |  |  |  |  |  | BAPQ | CATQ - assimilation | 0.817 |
| Scheerer et al. (2020) ^a^ | 176 | Female | Non-autistic | 19.7 (1.88) | • Canada |  |  |  | AQ | CATQ - masking | 0.233 |
|  | 176* | Female | Non-autistic | 19.7 (1.88) |  |  |  |  | AQ | CATQ - compensation | 0.453 |
|  | 176* | Female | Non-autistic | 19.7 (1.88) |  |  |  |  | AQ | CATQ - assimilation | 0.662 |
|  | 71 | Male | Non-autistic | 20.9 (2.92) |  |  |  |  | AQ | CATQ - masking | 0.032 |
|  | 71* | Male | Non-autistic | 20.9 (2.92) |  |  |  |  | AQ | CATQ - compensation | 0.267 |
|  | 71* | Male | Non-autistic | 20.9 (2.92) |  |  |  |  | AQ | CATQ - assimilation | 0.563 |
| Somerville et al. (2024) ^a^ | 32 | Male | Non-autistic | 47.55 (17.1) | • UK |  |  |  | AQ | CATQ - masking | 0.097 |
|  | 32* | Male | Non-autistic | 47.55 (17.1) |  |  |  |  | AQ | CATQ - compensation | 0.527 |
|  | 32* | Male | Non-autistic | 47.55 (17.1) |  |  |  |  | AQ | CATQ - assimilation | 0.646 |
|  | 72 | Female | Non-autistic | 39.03 (15) |  |  |  |  | AQ | CATQ - masking | 0.483 |
|  | 72* | Female | Non-autistic | 39.03 (15) |  |  |  |  | AQ | CATQ - compensation | 0.796 |
| Somerville et al. (2024) ^a^ *continued* | 72* | Female | Non-autistic | 39.03 (15) |  |  |  |  | AQ | CATQ - assimilation | 0.745 |
|  | 6 | Other | Non-autistic | 43.6 (13.8) |  |  |  |  | AQ | CATQ - masking | 0.287 |
|  | 6* | Other | Non-autistic | 43.6 (13.8) |  |  |  |  | AQ | CATQ - compensation | -0.027 |
|  | 6* | Other | Non-autistic | 43.6 (13.8) |  |  |  |  | AQ | CATQ - assimilation | 0.732 |
| Tamura et al. (2024) ^b^ | 146 | Male | Autistic |  | • Japan  • Age: 37.5 years; age of diagnosis: 31.8 years; | PHQ-9 | GAD-7 | LSAS | AQ-10 | CATQ - masking | -0.133 |
|  | 146* | Male | Autistic |  |  | PHQ-9 | GAD-7 | LSAS | AQ-10 | CATQ - compensation | -0.082 |
|  | 146* | Male | Autistic |  |  | PHQ-9 | GAD-7 | LSAS | AQ-10 | CATQ - assimilation | 0.195 |
|  | 120 | Female | Autistic |  |  | PHQ-9 | GAD-7 | LSAS | AQ-10 | CATQ - masking | 0.035 |
|  | 120* | Female | Autistic |  |  | PHQ-9 | GAD-7 | LSAS | AQ-10 | CATQ - compensation | 0.026 |
|  | 120* | Female | Autistic |  |  | PHQ-9 | GAD-7 | LSAS | AQ-10 | CATQ - assimilation | 0.215 |
|  | 21 | Other | Autistic |  |  | PHQ-9 | GAD-7 | LSAS | AQ-10 | CATQ - masking | 0.127 |
|  | 21* | Other | Autistic |  |  | PHQ-9 | GAD-7 | LSAS | AQ-10 | CATQ - compensation | 0.129 |
|  | 21* | Other | Autistic |  |  | PHQ-9 | GAD-7 | LSAS | AQ-10 | CATQ – assimilation | 0.441 |
| Tassone (2024) ^a^ | 152 | Male | Autistic (including self-diagnosed) | 25.8 (11.94) | • USA  • Diagnosis: formal ASD – 54%;  • Age of diagnosis: 21.27 years;  • Gender: female – 255, male – 251, transgender / non-binary – 250. |  |  |  | AQ-10 | CATQ - masking | 0.025 |
| Tassone (2024) ^a^ *continued* | 152* | Male | Autistic (including self-diagnosed) | 25.8 (11.94) |  |  |  |  | AQ-10 | CATQ - compensation | 0.234 |
|  | 152* | Male | Autistic (including self-diagnosed) | 25.8 (11.94) |  |  |  |  | AQ-10 | CATQ – assimilation | 0.524 |
|  | 255 | Female | Autistic (including self-diagnosed) | 35.02 (10.71) |  |  |  |  | AQ-10 | CATQ - masking | 0.024 |
|  | 255* | Female | Autistic (including self-diagnosed) | 35.02 (10.71) |  |  |  |  | AQ-10 | CATQ - compensation | 0.376 |
|  | 255 | Female | Autistic (including self-diagnosed) | 35.02 (10.71) |  |  |  |  | AQ-10 | CATQ – assimilation | 0.414 |
|  | 250 | Other | Autistic (including self-diagnosed) | 28.87 (8.1) |  |  |  |  | AQ-10 | CATQ - masking | -0.075 |
|  | 250* | Other | Autistic (including self-diagnosed) | 28.87 (8.1) |  |  |  |  | AQ-10 | CATQ - compensation | 0.215 |
|  | 250* | Other | Autistic (including self-diagnosed) | 28.87 (8.1) |  |  |  |  | AQ-10 | CATQ – assimilation | 0.352 |
| van der Putten et al. (2023) | 352 |  | Autistic | 52.3 (12.5) | • Netherlands  • 183 male, 167 female, 2 other; • Co-occurring condition: any mental health condition – 41%, any mood condition – 10.6%, any anxiety condition – 37.3%, other conditions – 6%. |  |  |  | AQ | CATQ | 0.29 |
| Walsh et al. (2023) ^b^ | 24 | Female | Autistic | 39.42 (13.2) | • USA |  |  |  | SRS-2 | CATQ - masking | 0.113 |
| Walsh et al. (2023) ^b^ *continued* | 24* | Female | Autistic | 39.42 (13.2) |  |  |  |  | SRS-2 | CATQ - compensation | 0.372 |
|  | 24* | Female | Autistic | 39.42 (13.2) |  |  |  |  | SRS-2 | CATQ - assimilation | 0.458 |
|  | 24* | Female | Autistic | 39.42 (13.2) |  |  |  |  | ADOS-2 Module 4 | CATQ - masking | 0.01 |
|  | 24* | Female | Autistic | 39.42 (13.2) |  |  |  |  | ADOS-2 Module 4 | CATQ - compensation | -0.094 |
|  | 24* | Female | Autistic | 39.42 (13.2) |  |  |  |  | ADOS-2 Module 4 | CATQ - assimilation | -0.108 |
|  | 24* | Female | Autistic | 39.42 (13.2) |  |  |  |  | ADOS-2 Module 4 | SRS-2 discrepancy | -0.191 |
|  | 24* | Female | Autistic | 39.42 (13.2) |  |  |  |  | SRS-2 | ADOS-2 Module 4 discrepancy | 0.992 |
|  | 21 | Male | Autistic | 41.95 (11.4) |  |  |  |  | SRS-2 | CATQ - masking | -0.084 |
|  | 21* | Male | Autistic | 41.95 (11.4) |  |  |  |  | SRS-2 | CATQ - compensation | 0.184 |
|  | 21* | Male | Autistic | 41.95 (11.4) |  |  |  |  | SRS-2 | CATQ - assimilation | 0.833 |
|  | 21* | Male | Autistic | 41.95 (11.4) |  |  |  |  | ADOS-2 Module 4 | CATQ - masking | 0.069 |
|  | 21* | Male | Autistic | 41.95 (11.4) |  |  |  |  | ADOS-2 Module 4 | CATQ - compensation | -0.009 |
|  | 21* | Male | Autistic | 41.95 (11.4) |  |  |  |  | ADOS-2 Module 4 | CATQ - assimilation | -0.213 |
|  | 21* | Male | Autistic | 41.95 (11.4) |  |  |  |  | ADOS-2 Module 4 | SRS-2 discprepancy | 0.354 |
| Walsh et al. (2023) ^b^ *continued* | 21* | Male | Autistic | 41.95 (11.4) |  |  |  |  | SRS-2 | ADOS-2 Module 4 discrepancy | 0.995 |
|  | 21 | Female | Non-autistic | 43.57 (15.7) |  |  |  |  | SRS-2 | CATQ - masking | 0.382 |
|  | 21* | Female | Non-autistic | 43.57 (15.7) |  |  |  |  | SRS-2 | CATQ - compensation | 0.519 |
|  | 21* | Female | Non-autistic | 43.57 (15.7) |  |  |  |  | SRS-2 | CATQ - assimilation | 0.622 |
|  | 19 | Male | Non-autistic | 48.53 (13.4) |  |  |  |  | SRS-2 | CATQ - masking | -0.124 |
|  | 19* | Male | Non-autistic | 48.53 (13.4) |  |  |  |  | SRS-2 | CATQ - compensation | -0.008 |
|  | 19* | Male | Non-autistic | 48.53 (13.4) |  |  |  |  | SRS-2 | CATQ – assimilation | 0.352 |
| Weiner et al. (2023) | 28 | Female | Autistic | 29.51 (8.01) | • France  • Age: 29.55 years; age of diagnosis: 26.41 years; • Autistic female: • DSM-5 comorbidities: BPD – 11%, ADHD – 43%, anxiety disorders – 32%, depression – 29%, BD – 4%, ED – 7%; • Self-harming behaviours: 93%; suicidal ideation: 93%, history of suicide attempts: 54%; • Autistic male: • DSM-5 comorbidities: ADHD – 41%, anxiety disorders – 9%, depression – 9%, BD – 4.5%; • Self-harming behaviours: 82%; suicidal ideation: 86%, history of suicide attempts: 54.5%. | BDI | BAI |  | AQ | CATQ | -0.039 |
|  | 22 | Male | Autistic | 31.64 (10.9) |  | BDI | BAI |  | AQ | CATQ | 0.226 |
| Wu et al. (2024) | 120 |  | Non-autistic | 35.42 | • UK  • High trait group:  • Age: 21.97 years; • Gender: female – 67.2%, male – 32.8%; • Autistic mother group:  • Age: 42.55 years; • Non-autistic mother group: • Age: 41.73 years; | BDI | STAI Y-2 |  | AQ | CATQ | 0.59 |

*Note.* *Same sample within study. ^a^ Authors provided additional data. ^b^ Authors provided dataset. All studies are cross-sectional unless otherwise noted. AQ = Autism Quotient, AQ-28 = Autism Quotient 28 items. AQ-S = Autism Quotient Short Form. AQ-10 = Autism Quotient – 10 Items. AQ (SOC+COM) = Autism Quotient (Social and Communication Subscales). BAPQ = Broad Autistic Phenotype Questionnaire. SRS-2 = Social Responsiveness Scale, 2^nd^ Edition. CATI = Comprehensive Autistic Trait Inventory. ADOS-2 = Autism Diagnostic Observation Schedule, Second Edition. ADOS-2 Module 4 = Autism Diagnostic Observation Schedule, Second Edition, Module 4: Revised Algorithm and Standardized Severity Scores. ADOS-2 Module 3&4 = Autism Diagnostic Observation Schedule, Second Edition, Module 3&4. RAADS-14 = Ritvo Autism Asperger Diagnostic Schedule – 14. CATQ = Camouflaging Autistic Traits Questionnaire. CC = Compensation Checklist. PHQ-9 = Patient Health Questionnaire-9. DASS-21 = Depression, Anxiety, and Stress Scales – 21 items. BDI = Beck’s Depression Inventory. BDI-II = Beck’s Depression Inventory-II. CES-D = The Centre for Epidemiologic Studies Depression Scale. GAD-7 = General Anxiety Disorder-7. BAI = Beck Anxiety Inventory. STAI Y-2 = State-Trait Anxiety Inventory Form Y-2. ASA-A = Anxiety Scale for Autism-Adults. RCADS = Revised Children’s Anxiety and Depression Scale. ASD = Autism Spectrum Disorder. AS = Asperger’s Syndrome. HFA = High Functioning Autism. PDD = Pervasive Development Disorder. PDD-NOS = Pervasive Developmental Disorder – Not Otherwise Specified. IDD = Intellectual and Developmental Disabilities. LD = Learning Disability. LD = Specific Learning Disorder. BPD = Borderline Personality Disorder. BD = Bipolar Disorder. ADHD = Attention Deficit and Hyperactivity Disorder. ED = Eating Disorder. SAD = Social Anxiety Disorder. GAD = Generalised Anxiety Disorder. OCD = Obsessive Compulsive Disorder. PTSD = Post Traumatic Stress Disorder. MDS = Major Depressive Symptoms.
