# Supplementary material for: Autistic Traits and Camouflaging: A Meta-Analysis
Source: Autism. 2026 Apr 24;30(6):1398–415. doi: 10.1177/13623613261437500 (PMC13187235; doi:10.1177/13623613261437500)
Supplement: sj-docx-3-aut-10.1177_13623613261437500 – Supplemental material for Autistic Traits and Camouflaging: A Meta-Analysis [file sj-docx-3-aut-10.1177_13623613261437500.docx]

**Supplementary 3**

**4.1. Gender Moderation**

A separate sub-group analysis was conducted to test the effect of gender while also including a third gender-nonconforming group, however the model remained non-significant, *F*(2,246) = 1.24, *p* = .292; *Q* = 2968.93, *p* < .001, with a small to moderate effect for the gender non-conforming group, *r* = 0.26, 95% CI [0.07, 0.44], *k* = 14.

**4.2. Diagnostic Status Moderation**

A separate sub-group analysis was conducted to test the effect of diagnostic status while also including self-diagnosed participants in the autistic group. Diagnostic status was a significant moderator, *F*(1,261) = 14.46, *p* < .001; *Q* = 3929.12, *p* < .001. While the relationship was significant for autistic participants (*r*  = 0.26, 95% CI [0.18, 0.33], *k* = 57) and those in the general population (*r* = 0.45, 95% CI [0.37, 0.54, *k* = 45), the strength of this relationship increased from moderate to large for the general population.
